# Integrative spatial and single-cell transcriptomics elucidate programmed cell death-driven tumor microenvironment dynamics in hepatocellular carcinoma

**DOI:** 10.3389/fimmu.2025.1589563

**Published:** 2025-07-16

**Authors:** Kai Lei, Yutong Zhao, Shumin Li, Jiawei Liu, Wenhao Chen, Caihong Zhou, Yi Zhang, Jinmei Tan, Jian Wu, Qi Zhou, Jiehui Tan

**Affiliations:** ^1^ Center of Hepato-Pancreato-Biliary Surgery, The First Affiliated Hospital, Sun Yat-sen University, Guangzhou, Guangdong, China; ^2^ Department of General Surgery, Hui Ya Hospital of The First Affiliated Hospital, Sun Yat-sen University, Huizhou, Guangdong, China; ^3^ Department of Oncology, Cancer Center, The First Affiliated Hospital, Sun Yat-sen University, Guangzhou, Guangdong, China; ^4^ Department of Hepatobiliary Surgery, The Third Affiliated Hospital, Sun Yat-sen University, Guangzhou, Guangdong, China; ^5^ Division of Hepatobiliopancreatic Surgery, Department of General Surgery, Nanfang Hospital, Southern Medical University, Guangzhou, Guangdong, China; ^6^ Department of Intensive Care Unit, Wuchuan People’s Hospital, Zhanjiang, Guangdong, China

**Keywords:** hepatocellular carcinoma, programmed cell death, prediction model, tumor microenvironment, Ube2E1

## Abstract

**Purpose:**

Programmed cell death (PCD) mechanisms play crucial roles in cancer progression and treatment response. This study aims to develop a PCD scores prediction model to evaluate the prognosis of hepatocellular carcinoma (HCC) and elucidate the tumor microenvironment differences.

**Methods:**

We analyzed transcriptomic data from 363 HCC patients in the TCGA database and 221 patients in the GEO database to develop a PCD prediction model. Single-cell RNA sequencing (scRNA-seq) and spatial transcriptomics sequencing (ST-seq) data from HCC patients were analyzed to investigate the tumor microenvironment and functional disparities. The oncogenic role of the key gene UBE2E1 in the model was explored in HCC through various *in vitro* experiments.

**Results:**

Seventeen PCD-related genes were identified as significant prognostic indicators, forming the basis of our PCD prediction model. High-PCD scores correlated with poorer overall survival (OS) and exhibited significant predictive capabilities. scRNA-seq analysis revealed distinct tumor cell characteristics and immune microenvironment differences between high- and low-PCD groups. High-PCD tumors showed increased cell proliferation and malignancy-associated gene expression. T cells in high-PCD patients were more likely to be exhausted, with elevated expression of exhaustion markers. ST-seq data also confirmed these results. Among the genes associated with the PCD prognostic model, UBE2E1 was identified as a key oncogenic marker in HCC.

**Conclusions:**

The PCD prediction model effectively predicts prognosis in HCC patients and reveals critical insights into the tumor microenvironment and immune cell exhaustion. This study underscores the potential of PCD-related biomarkers in guiding personalized treatment strategies for HCC.

## Introduction

Liver cancer ranks as the sixth most prevalent cancer globally and stands as the third highest cause of cancer-related deaths, preceded only by lung and colorectal cancer (1). Hepatocellular carcinoma (HCC) emerges as the predominant form of primary liver cancer. Over the past two decades, substantial progress in systemic therapies has markedly improved the prognosis for advanced-stage HCC patients (2–4). However, the high heterogeneity of cancer remains a central challenge, complicating the search for effective therapeutic strategies (5). This problem is not limited to HCC; similar issues are faced by other cancer types, such as muscle-invasive bladder cancer (MIBC), where neoadjuvant combination therapies have shown promising efficacy, with HER2 and HSPA1A expression emerging as potential biomarkers for treatment response (6, 7). Personalized treatment plans tailored to individual patient conditions are therefore imperative. Additionally, identifying reliable biomarkers for high-risk populations is crucial for enhancing diagnostic accuracy, treatment specificity, and prognosis. These efforts aim to broaden the spectrum of strategies available to combat cancer.

Resisting cell death is a hallmark of cancer, pivotal at various stages of its progression (8). Evading cell death is a key characteristic of cancer. Cell death can be classified into two categories based on different triggering stimuli: accidental cell death (ACD) and programmed cell death (PCD). ACD occurs spontaneously due to severe physical, chemical, or mechanical damage, while PCD relies on specialized molecular mechanisms and can be modulated pharmacologically or genetically (9, 10). Fifteen types of programmed cell death (PCD) have been identified, including: anoikis (ANK), apoptosis-like morphology (ALM), autophagy (ATG), cuproptosis (CUP), entotic cell death (ENT), extrinsic apoptosis (EAP), ferroptosis (FPT), immunogenic cell death (ICD), intrinsic apoptosis (IAP), lysosome-dependent cell death (LDC), necroptosis (NPT), necrosis-like morphology (NLM), necrosis (NCR), parthanatos (PRT), and pyroptosis (PYR) (9, 11–13).

Recent studies highlight the significance of PCD mechanisms in liver cancer treatment. Pyroptosis, mediated by gasdermin E (GSDME), is a promising molecular mechanism for chemotherapy drugs in tumor treatment, particularly in liver cancer. This process inhibits tumor growth and induces apoptosis, offering potential therapeutic benefits (14). Additionally, intra-tumor NF-κB-pyroptosis correlates with poor prognosis in liver cancer patients. Inactivation of NF-κB has been shown to accelerate necroptosis, thereby mitigating inflammation and liver cancer progression (15). Moreover, ferroptosis has been implicated in sorafenib resistance, radioresistance, and immune evasion in HCC (16–18).

Given the fundamental role of various PCD mechanisms in the development and progression of HCC, we propose a novel approach utilizing PCD-related molecules to establish a predictive indicator for PCD risk scoring, which is crucial for determining the prognosis of HCC patients. Leveraging single-cell RNA sequencing (scRNA-seq) technology and spatial transcriptomics sequencing (ST-seq) techniques, we aim to delineate the cellular composition of the tumor microenvironment and discern functional disparities in tumor cells between high-risk and low-risk patients. Additionally, we intend to conduct detailed analyses of T cells using scRNA-seq and ST-seq technology, thereby shedding new light on how PCD influences the prognosis of HCC patients. Most notably, we elucidated the oncogenic function of the model key gene UBE2E1 in HCC through *in vitro* experimentation.

## Materials and methods

### Data collection

Essential genes associated with 15 PCD patterns were compiled from various sources. We sourced the essential genes from different established databases and publications, including the Kyoto Encyclopedia of Genes and Genomes (KEGG) for comprehensive genetic information, the Molecular Signatures Database (MSigDB, accessible at http://software.broadinstitute.org/gsea/msigdb/index.jsp), various peer-reviewed articles, and a meticulously curated collection from the Genecards website (refer to https://www.genecards.org/ for detailed genetic profiles) (10, 11, 19). Our comprehensive analysis encompassed a range of genes, including 27 genes in anoikis, 146 genes in apoptosis-like morphology, 222 genes in autophagy, 17 genes in cuproptosis, 23 genes in entotic cell death, 500 genes in extrinsic apoptosis, 283 genes in ferroptosis, 500 genes in immunogenic cell death, 500 genes in intrinsic apoptosis, 194 genes in lysosome-dependent cell death, 500 genes for necroptosis, 73 genes involved in necrosis-like morphology, 500 genes in necrosis, 23 related to parthanatos, and 388 associated with pyroptosis (**Supplementary Table S1**). After the removal of duplicates, we incorporated a total of 2159 unique PCD-related genes into our analysis. Transcriptomic data of the TCGA cohort was downloaded from the University of California Santa Cruz (UCSC) Xena data portal (https://xenabrowser.net) (20). After excluding duplicate samples, samples lacking clinical information, and samples with a survival time of zero, 363 HCC samples were ultimately included in the study. Additionally, we obtained microarray data and clinical characteristics (GSE14520) from the Gene Expression Omnibus (GEO) database (https://www.ncbi.nlm.nih.gov/geo/).

To address the technical heterogeneity between the RNA-seq (TCGA) and microarray (GEO) platforms, we applied Z-score normalization to the expression matrix of each cohort individually. Additionally, to remove batch effects and harmonize data distributions across platforms for model application, we used the ComBat function from the sva R package (version 3.48.0), following standard procedures. Genes were intersected between platforms before normalization to ensure compatibility. This preprocessing allowed for consistent calculation and application of the PCD-based scoring system across cohorts.

ScRNA-seq data of HCC patients were collected from GSE151530, GSE125449, and GSE149614. The ST-seq data were collected from HRA000437 (https://ngdc.cncb.ac.cn/gsa-human/browse/HRA000437).

### Construction of the PCD scores prediction model

Initially, through univariate Cox analysis, we preliminarily identified PCD-related genes with prognostic value. To avoid omission, we set the cut-off *p-*value at 0.1. Further, multivariate Cox analysis was employed to determine PCD genes of prognostic significance, with the cut-off *p*-value set at 0.05. Subsequently, the LASSO was utilized to establish a penalty function, and a more precise model was obtained through the “glmnet” package (21). Ultimately, the model outputted the PCD score for each patient using the following formula:


$$PCD\;score=\;\overset{n}{\underset{i=1}{\sum }}Coef\left(\beta i\right)*Exp\left(Xi\right)$$

In the formula, Coef (βi) represents the risk coefficient, and Exp (χi) denotes the expression of each gene, with n indicating the number of genes in the model. The PCD score cut-off value in each cohort was defined as the median score within that cohort to ensure internal consistency and avoid information leakage across datasets. Full PCD score details and stratification information for each sample are provided in **Supplementary Tables S3**–**S5**. We used the “survival” and “survminer” packages to perform Kaplan-Meier analysis to investigate the correlation between overall survival (OS) time and PCD. Additionally, Receiver Operating Characteristic (ROC) curves have been generated to assess the prognostic efficacy of the PCD.

### scRNA-seq data preprocessing

The unique molecular identifier (UMI) count matrix was converted into a Seurat object using the R package “Seurat” (version 4.3.0). Subsequent quality control was conducted, excluding low-quality cells based on the following criteria: samples with fewer than 500 cells and cells with fewer than 300 detected genes were excluded, and the percentage of mitochondrial genes was maintained below 15%. “DoubletFinder” was utilized to identify potential doublets within the samples. The expected doublet rate was adjusted to align with the Poisson doublet formation rate, which was determined through a calculation that took into account cell concentration (22). Following quality control, a dataset comprising 41,301 cells and 25,714 genes was obtained for downstream analysis.

### Unsupervised cell clustering, marker Identification, and differential gene expression

The Seurat (version 4.3.0) R package was utilized for the analysis of scRNA-seq data (23). We normalized the expression measurements of each cell’s features relative to their total expression levels using the ‘LogNormalize’ methodology. First, we scaled the data by multiplying it by a factor of 10,000. Then, we performed a logarithmic transformation on the scaled results. Based on the normalized gene expression matrix, we identified 2,000 highly variable genes using the “FindVariableFeatures” function and the variance stabilizing transformation (VST) method. Subsequently, we performed Principal Component Analysis (PCA) on these genes with significant variation. The number of principal components (PCs) suitable for downstream analysis was determined using an Elbowplot, which was set to 35 in this case. We used the default parameters of Harmony (version 0.1.1) to correct batch effects (24). Subsequently, we performed graph-based clustering using shared nearest neighbor (SNN) and the Louvain graph-based algorithm. We implemented clustering using the FindNeighbors and FindClusters functions of the Seurat package. The t-distributed Stochastic Neighbor Embedding (t-SNE) and Uniform Manifold Approximation and Projection (UMAP) were utilized to visualize the clustering results.

To better define distinct clusters, we identified differential genes using the FindAllMarkers function from the Seurat R package. Only genes with a log fold-change greater than 0.25 between the two groups and detectable expression in more than 25% of cells in either population were considered differentially expressed. Subsequently, we annotated the clusters based on the expression profiles of known canonical marker genes. The marker genes for each cluster are presented in **Supplementary Table S2**.

### Copy number variation analysis using CopyKAT

To further distinguish malignant tumor cells from non-malignant populations, we performed CNV inference using CopyKAT (v1.1.0) (25). CopyKAT applies integrative Bayesian approaches on single-cell RNA-seq data to identify large-scale chromosomal copy number aberrations and distinguish aneuploid tumor cells from diploid normal cells. Given that all samples were derived from tumor tissue, we used T cells, which are typically diploid and genetically stable, as a reference population for CNV analysis. CopyKAT was run following the standard pipeline and parameters as described in the official tutorial. The results provided genome-wide CNV profiles, allowing us to confirm the malignancy of cells annotated as tumor subpopulations based on gene expression.

### Calculation and grouping of PCD scores for single-cell samples

We aggregated the gene counts of all cells using the AggregateExpression function in the Seurat R package, resulting in a gene expression profile for each sample. Subsequently, we calculated the risk value for each sample using a well-established formula and grouped them accordingly. Specifically, the top 14 samples were defined as the high PCD risk group, while the bottom 14 samples were designated as the low PCD risk group.

### Differentially expressed genes and pathway enrichment analysis

To evaluate the biological functional differences within each cell type (tumor cells and T cells) between the high-PCD group and the low-PCD group, we conducted differential expression analysis and pathway enrichment analysis. The analysis was based on the Wilcoxon Rank-Sum test using the “FindMarkers” function in the Seurat package. DEGs were defined with an adjusted *p-*value threshold of 0.05. These DEGs were then utilized in the clusterProfiler R package (version 4.8.3) for KEGG pathway enrichment analysis (26–28). Pathways with an adjusted *p-*value< 0.05 were considered significantly enriched.

### Metabolic pathway activity analysis

The weighted relative pathway activity algorithm was employed to assess the metabolic pathway activity in both high-PCD and low- PCD groups (29). For each cell type, we calculated the average expression level of metabolic genes pertinent to that cell type. This was followed by a comparison of each gene’s expression level in a given cell type with its average expression across all cells, yielding the relative expression level. For every metabolic pathway, we computed the weighted average of the relative expression levels of the genes constituting that pathway. To reduce the impact of genes with either low expression levels or high missing data rates on the pathway activity assessment, outliers were excluded-specifically those with relative expression levels exceeding three times the 75th percentile or falling below one-third of the 25th percentile in each pathway.

A random permutation test was utilized to evaluate the statistical significance of the pathway activity in distinct cell types. By randomly shuffling cell labels 5,000 times, we simulated a null distribution of the pathway activity scores. These scores were then contrasted with those in the original, non-rearranged dataset to compute *p-*values.

### Cellchat analysis

We used the cellchat R package (version 1.6.1) to infer cell communication between different groups of tumor cells and all types of T cells (30). Following the official workflow, we constructed CellChat objects for the two groups of single cells using the “createCellChat” function. Subsequently, the “computeCommunProb” function was employed to infer communication probabilities. To ensure robust analysis of cell-cell communication within our dataset, we further filtered out interactions where fewer than ten cells per cell type were involved, using the “filterCommunication” function. This rigorous approach allowed us to focus on the most reliable and biologically meaningful cellular interactions.

### Developmental trajectory inference

To investigate the functional variations and potential lineage differentiations among CD8+ T cells, we employed the “Monocle2” R package (version 2.28.0) to reconstruct the cellular differentiation trajectory of CD8+ T cell subsets (31). The single-cell expression matrix data of CD8+ T cells were converted into the CellDataSet class using the newCellDataSet function within the “Monocle2” R package for subsequent analysis. Subsequently, we utilized the VariableFeatures function to identify highly variable genes, which were used to define the cellular processes. DDRTree within the reduceDimension function was applied for data dimensionality reduction. Based on the Reversed Graph Embedding algorithm, cells were arranged in pseudotime order along the trajectory.

### Spatial transcriptomics integration and risk score assessment

Given the current ST resolution, each spot is estimated to contain approximately 8 to 20 cells, which precludes the assignment of specific cell types to each spot. To elucidate the cellular composition of each spot, we integrated HCC single-cell data with spatial transcriptomics data using SPOTlight (32). SPOTlight employs non-negative matrix factorization (NMF) regression to derive cell type-specific gene signatures.

Subsequently, we defined spots with the highest tumor proportion as “malignant regions”, while the remaining spots were categorized as “other regions”. By calculating the risk score for each spot, we identified the top and bottom 5% of spots with the highest and lowest risk scores, respectively. The AUCell R package was then utilized to evaluate the characteristics of each group of spots (33).

### Differential expression and prognostic association of UBE2E1 in HCC

Using the GEPIA2 (http://gepia2.cancer-pku.cn/) database, we verified the differential expression of UBE2E1 between HCC samples and peri-tumor as well as normal samples. Kaplan-Meier analysis was performed to calculate survival estimates, and a Kaplan-Meier survival curve was generated.

### Cell lines and culture

Human HCC Huh-7 cells Huh7 cells and SNU-449 cells were obtained from the China Center for Type Culture Collection (Shanghai, China). Huh-7 cells were cultured in Dulbecco’s modified Eagle’s medium (DMEM, Gibco) supplemented with 10% fetal bovine serum (GIBCO, USA) and 1% penicillin-streptomycin (GIBCO, USA). The cells were grown in a 5% CO_2_ incubator (Thermo Scientific, USA) at 37°C.

### Knockdown of UBE2E1 in HCC cells

The pLKO.1 lentiviral vectors expressing short hairpin RNA (shRNA) targeting UBE2E1 were purchased from Umine Biotechnology Co., LTD (China). The shRNA constructs, along with the packaging plasmid (pCMV-ΔR8.9) and the envelope plasmid (pCMV-VSVG), were co-transfected into HEK293T cells using Lipofectamine 3000 (Invitrogen, USA). After 48 hours of incubation, the viruses were collected and used to infect Huh-7 cells with polybrene (8 μg/ml) (Solarbio, China). Stable infected cells were then selected with puromycin (2.5 μg/ml) for 48 hours. The shRNA sequences used in this study are as follows: shUBE2E1-1: CCCAAGAAGAAGGAGAGTAAA; shUBE2E1-2: CTTGGTAAAGAGTAGGGTATT.

### Western blot

After homogenization and centrifugation, the supernatant was collected for total protein quantification using the BCA protein assay kit (Solarbio, China). Fifty micrograms of protein from each sample were loaded onto sodium dodecyl sulfate-polyacrylamide gel electrophoresis (SDS-PAGE), followed by transfer to polyvinylidene difluoride (PVDF) membranes. Nonspecific binding sites were blocked with 5% bovine serum albumin (BSA) prior to antibody incubation. The membranes were then incubated with primary antibodies (Proteintech, China) overnight at 4°C. Subsequently, the membranes were washed with TBS and incubated with horseradish peroxidase (HRP)-conjugated secondary antibodies in blocking buffer for 1 hour at room temperature. After three washes, the protein bands were visualized using an enhanced chemiluminescence detection kit (Solarbio, China).

### Clone formation, cell proliferation, viability and migration assays

In the clonogenic assay, 1000 cells/well were seeded into six-well plates containing fresh medium. After 2 weeks, colonies were stained with 0.5% crystal violet and counted. For viability assessment, 2000 cells/well were plated in 96-well plates with fresh medium. Cell viability was evaluated at 6, 24, 48, 72, 96 and 120 hours using the Cell Counting Kit-8 (Dojindo, Japan) according to the manufacturer’s instructions.

A total of 5 × 10^4 cells suspended in 500 μl of serum-free fresh medium were added to the upper chamber of transwell inserts (Corning Falcon, USA), which were then placed in wells containing fresh medium with 20% fetal bovine serum to induce cell migration. For the migration assay, an equal number of cells were seeded into chambers. After 48 hours, cells that had migrated to the outer surface were stained with 0.5% crystal violet and counted to analyze cell migration.

### Determination of apoptosis

Huh7 cells and SNU-449 cells were seeded into six-well plates. Apoptosis was tested using an apoptosis detection kit according to the manufacturer’s instructions (Dojindo, Japan). Cells in the six-well plates were collected and cell subpopulations were analyzed. Initially, cells were gated based on forward scatter (FSC−) versus side scatter (SSC−) characteristics. Subsequently, doublets were excluded using consecutive gating on FSC-Area versus FSC-Width and SSC-Area versus SSC-Width plots. Quadrants were set on the Annexin V/PI dot plot. Living cells (Annexin V−/PI−), early apoptotic cells (Annexin V+/PI−), late apoptotic cells (Annexin V+/PI+), and necrotic cells (Annexin V−/PI+) were distinguished. Afterwards, the proportions of annexin V+/PI−, annexin V+/PI+, annexin V−/PI+, and annexin V−/PI− cell populations were calculated using quadrant gates. Data were analyzed using FlowJo v 9.6.3 (TreeStar, Inc).

### Animal model and tumor xenograft implantation

NCG mice (Strain No. T001475) were purchased from GemPharmatech. For the subcutaneous transplantation, a total of 10 mice were used, with 5 mice injected with Huh7 cells transfected with shNC (control) and 5 mice injected with Huh7 cells transfected with shUBE2E1. A total of 5 × 10^6 cells in 200 µL PBS were injected subcutaneously into the right flank of each mouse. After 2 weeks, the mice were euthanized, and the subcutaneous tumors were harvested. Tumor images were captured and tumor volume was measured for subsequent analysis. This methodology follows ethical guidelines for the use of animals in research and ensures that all procedures comply with approved protocols. The animal experiments were approved by the Institutional Animal Care and Use Committee (IACUC) at Sun Yat-sen University, under the Affidavit of Approval of Animal Use Protocol (Approval No. SYSU-IACUC-2025-001259).

### Statistical analysis

Statistical analyses were performed using R (version 4.1.2). Comparisons for categorical variables were conducted using the Chi-square test or Fisher’s exact test, while the Student’s t-test or Wilcoxon rank-sum test was employed for continuous variables. A *p-*value less than 0.05 was considered statistically significant.

## Results

### Study cohorts and workflow

**Figure 1:**
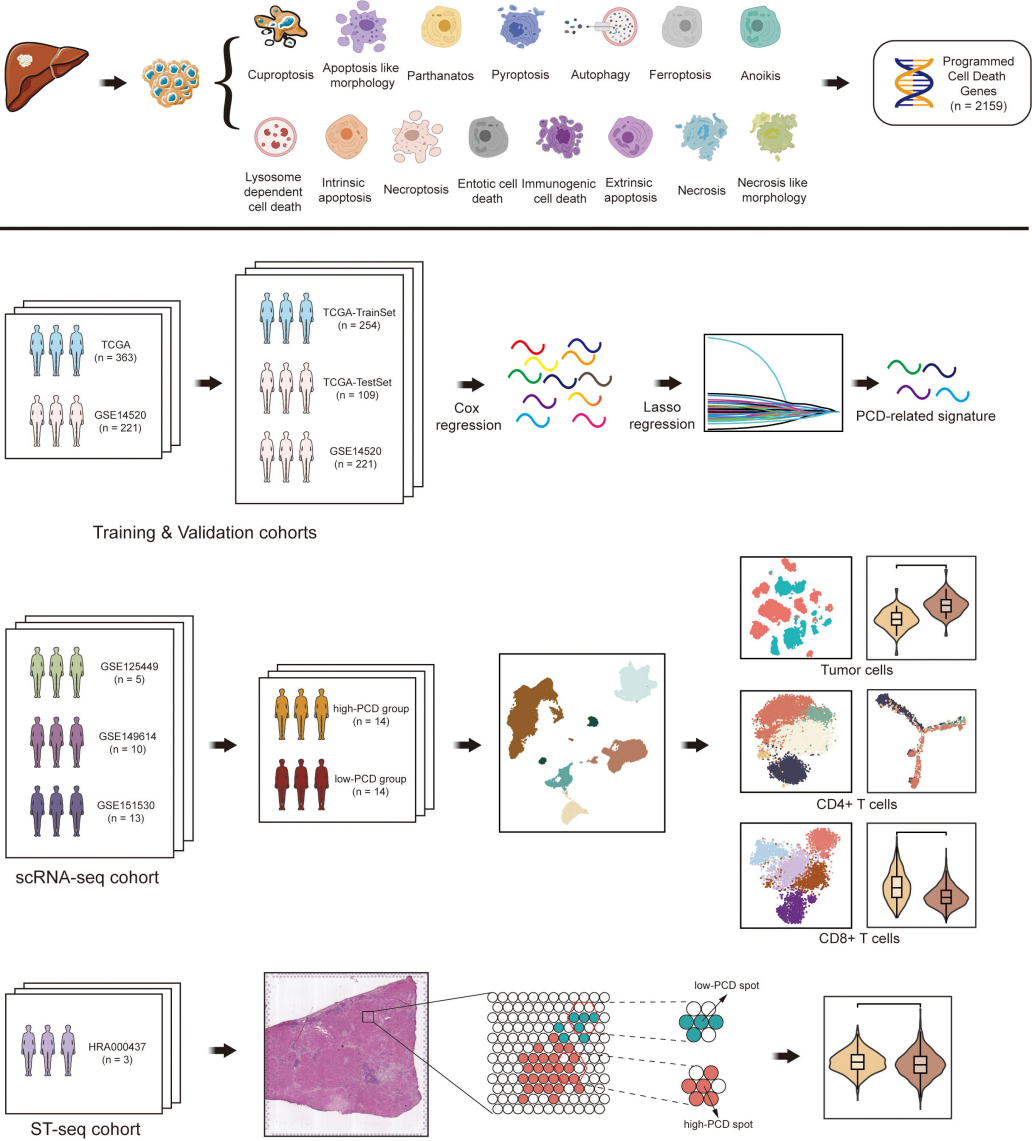
Flowchart illustrating the comprehensive analysis of diverse programmed cell death (PCD) patterns in hepatocellular carcinoma (HCC) patients.

In this study, we identified 363 hepatocellular carcinoma (HCC) patients from TCGA and 221 HCC patients from GSE14520 for training and validation cohorts, respectively. Among the 363 HCC patients from TCGA, a 7:3 random split was applied to allocate them into a training set (TCGA_TrainSet) and an internal validation set (TCGA_TestSet), while the 221 HCC patients from GSE14520 served as an external validation set. For the scRNA-seq cohort, 5 patients with HCC were collected from GSE125449, 10 patients from GSE149614, and 13 patients from GSE151530. For the spatial transcriptomics sequencing (ST-seq) cohort, the ST-seq data of 3 HCC patients were obtained from HRA000437. The workflow of this study is illustrated in **Figure 1**.

### Construction of a PCD scores prediction model for HCC patients

In the training set, univariate Cox regression analysis was utilized for the preliminary screening of genes associated with overall survival (OS) among the 2159 genes retrieved for 15 PCD patterns. Genes with a *p*-value less than 0.1 underwent further analysis using multivariate Cox regression related to OS. Subsequently, genes with a p-value less than 0.05 underwent LASSO regression to select the most predictive genes serving as OS-related indicators. Ultimately, 17 PCD-related genes (ENO1, CDK4, RPS17, PDLIM1, KLHDC10, IGFBP3, UBE2E1, CBS, UBB, YWHAQ, SPP1, USF2, STAT6, PCK2, CACYBP, HDAC1, CD79A) associated with OS were identified (**Figure 1** and **Supplementary Figure S1**). Among the 17 PCD-related genes, 1 gene originated from ATG (HDAC1), 5 genes from EAP (CDK4, IGFBP3, SPP1, STAT6, YWHAQ), 1 gene from FPT (PCK2), 3 genes from ICD (CD79A, CDK4, SPP1), 4 genes from IAP (CDK4, HDAC1, IGFBP3, YWHAQ), 3 genes from LDC (CDK4, UBB, UBE2E1), 2 genes from NPT (KLHDC10, RPS17), 2 genes from NLM (ENO1, PDLIM1), 3 genes from NCR (CBS, IGFBP3, SPP1), and 1 gene from PYR (USF2).

**Figure 2:**
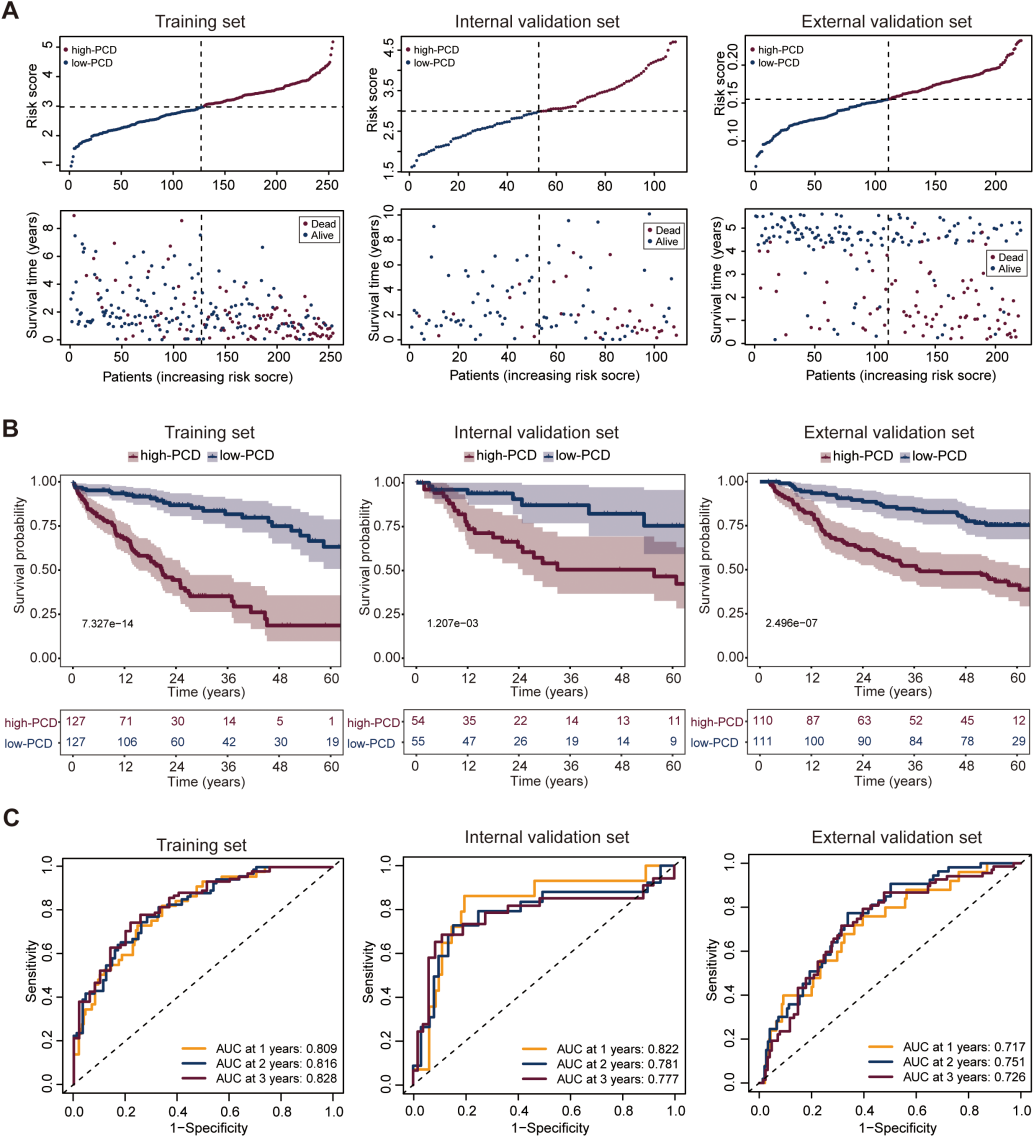
Development and validation of the PCD scores prediction model. **(A)** Distribution of PCD scores by survival time and survival status in the training set (TCGA_TrainSet), internal validation set (TCGA_TestSet), and external validation set (GSE14520). **(B)** Overall survival (OS) of HCC patients in the low- and high-PCD groups in the training set (TCGA_TrainSet), internal validation set (TCGA_TestSet), and external validation set (GSE14520). **(C)** Receiver Operating Characteristic (ROC) analysis for the PCD scores prediction model evaluating 1-, 2-, and 3-year OS in the training set (TCGA_TrainSet), internal validation set (TCGA_TestSet), and external validation set (GSE14520).

HCC patients in the TCGA_test cohort were stratified into high-PCD group (n = 127) and low-PCD group (n = 127) using the median of the PCD scores as the cut-off value. To compare the OS among HCC patients with varying PCD scores, the results revealed that individuals with a high PCD scores had a lower survival rate compared to those with a low PCD scores (**Figure 2A**). Furthermore, a notable disparity in OS was noted between these two groups, indicating that patients in the high-PCD group exhibited a higher probability of experiencing poorer OS outcomes (*p* = 7.327e-14) (**Figure 2B**). The receiver operating characteristic (ROC) curve analysis revealed that the PCD scores could assess 1-, 2-, and 3-year OS with an AUC value of 0.809, 0.816, and 0.828, respectively, indicating a significant predictive capability of the PCD scores (**Figure 2C**).

### Internal and external validation of the PCD scores prediction model

The patients with HCC in both the internal and external validation sets were similarly categorized into high-PCD and low-PCD groups using a predetermined cut-off derived from the median. When evaluating the predictive performance of the PCD scores in estimating OS among HCC patients, both internal and external validation sets demonstrated consistent trends. Within the internal validation set, patients with a high PCD score exhibited a markedly lower survival rate compared to those with a low PCD score, illustrating a substantial prognostic difference (**Figure 2A**). This observation was further supported by the pronounced contrast in OS outcomes between the high and low-PCD groups (*p* = 1.207e-03), suggesting a higher likelihood of adverse OS outcomes in patients with elevated PCD scores (**Figure 2B**). Similarly, the external validation set corroborated these findings, showing a consistent association between PCD scores and OS (**Figure 2A**). Patients with high PCD scores displayed a significantly inferior OS compared to those with low PCD valuse (*p* = 2.496e-07), reinforcing the robustness of the predictive model across different patient cohorts (**Figure 2B**).

The ROC curve analysis highlighted the predictive efficacy of the PCD scores prediction model, yielding AUC values of 0.822, 0.781, and 0.777 for 1-, 2-, and 3-year OS predictions, respectively, in the internal validation set (**Figure 2C**). In the external validation set, corresponding AUC values of 0.717, 0.751, and 0.726 were observed for 1-, 2-, and 3-year OS predictions (**Figure 2C**), affirming the robustness of the model across different validation cohorts.

### Profiling of tumor microenvironment in high- and low-PCD groups by scRNA-seq

To explore the detailed distinction of the tumor immune microenvironment in high- and low-PCD groups among HCC patients, we leveraged publicly available single-cell transcriptome datasets (GSE125449, GSE151530, and GSE149614) for analysis. Following quality control and filtering, a total of 71799 high-quality cells were obtained, with an average of 2564 cells per sample and an average detection of 25714 genes per cell. Based on typical individual marker genes, the total cells were partitioned into six major cell types using UMAP, including immune cells (T/NK cells, myeloid cells, B and plasma cells), tumor cells, and stromal cells (endothelial and fibroblasts cells) (**Supplementary Figure S2A**). Tumor cells were identified using *EPCAM*, *KRT18*, and *AFP* as markers, while T/NK cells were identified by using the markers *CD3D*, *CD3E*, and *GZMA* (**Supplementary Figure S2B**). To further validate the malignant identity of tumor cell subpopulations, we applied CopyKAT, which infers genome-wide large-scale copy number aberrations from single-cell transcriptomic profiles without requiring prior tumor annotations. The resulting UMAP embedding based on ploidy predictions revealed a distinct cluster of aneuploid cells (red), corresponding to the transcriptomically defined tumor population. In contrast, diploid cells (blue) were enriched in T/NK cell clusters, supporting their non-malignant identity. The clear segregation between aneuploid and diploid populations reinforces the validity of our tumor cell classification strategy (**Supplementary Figure S2C**). In the immune cell population, T cells comprise the largest proportion, with the proportion of T cells in the high-PCD group being significantly lower than that in the low-PCD group (**Supplementary Figure S2D**). Cell cycle states were identified among all available cells across the two groups, revealing a higher proportion of cells in the G2/M state in the high-PCD group compared to the low-PCD group, suggesting a greater degree of active cell proliferation in the high-PCD group (**Supplementary Figures S2E, F**).

### Distinguishing characteristics of tumor cells in high- and low-PCD groups

**Figure 3:**
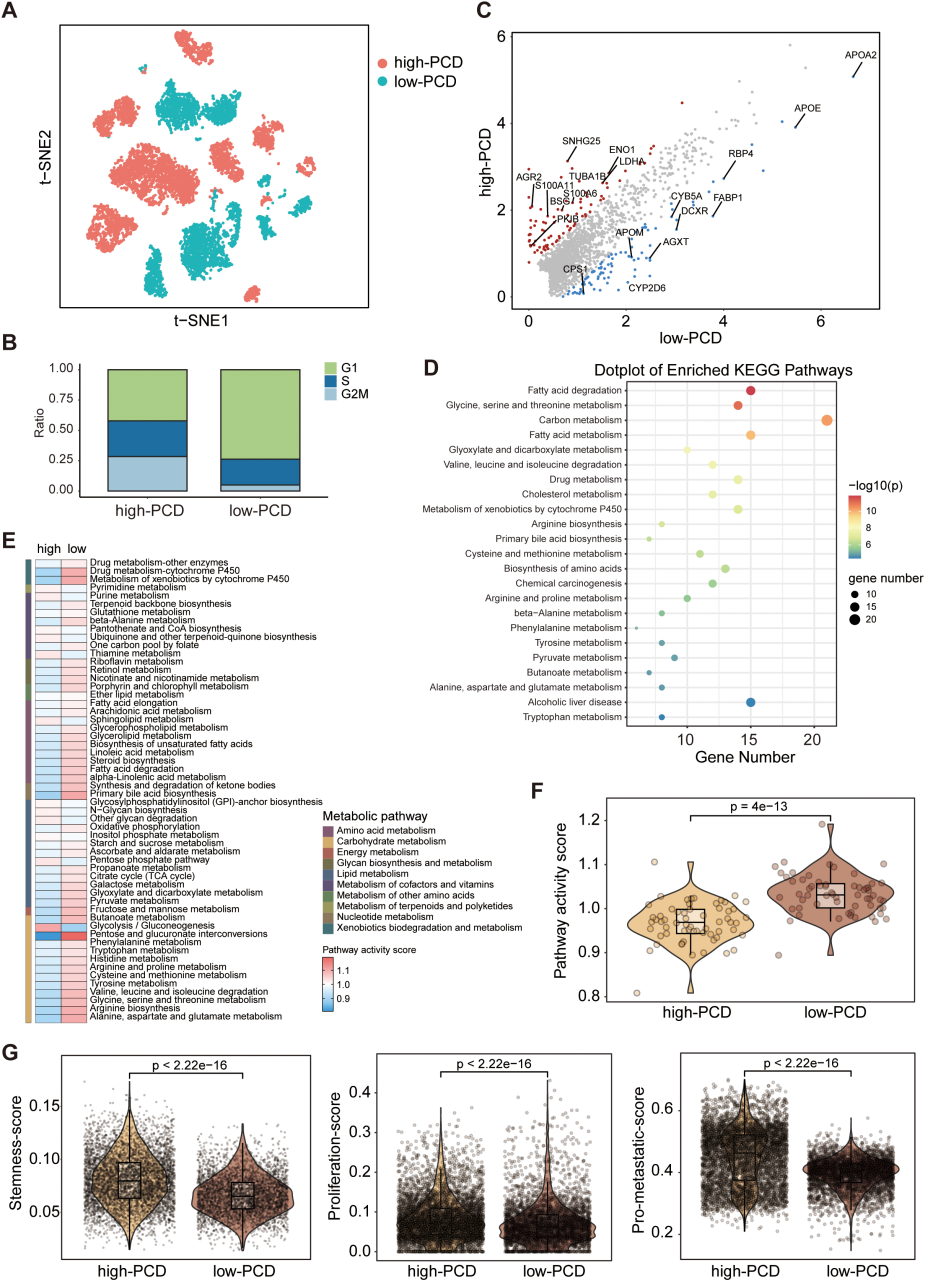
Deciphering the molecular characteristics of tumor cells in high-PCD and low-PCD groups based on scRNA-seq data. **(A)** The t-distributed stochastic neighbour embedding (t-SNE) plot of patients subgroups in tumors cells. **(B)** Proportions of cells with different cell cycle phases in high- and low-PCD groups. **(C)** jScatter plots depicting the significantly differentially expressed genes in tumor cells from high-PCD and low-PCD groups. **(D)** Kyoto Encyclopedia of Genes and Genomes (KEGG) pathway enrichment analysis in tumor cells in low-PCD group versus high-PCD group. Heatmap **(E)** and violin plot **(F)** illustrating the differences in metabolic pathway activity scores among tumor cells between the high-PCD and low-PCD groups; p value from the Wilcoxon test. **(G)** Violin plots displaying the expression scores of stemness, proliferation, and metastatic signatures in tumor cells from the high-PCD and low-PCD groups; p values from the Wilcoxon test.

To investigate the distinguishing tumor-intrinsic features between high- and low-PCD groups, a total of 9611 tumor cells were identified across 28 samples, comprising 5460 tumor cells in the high-PCD group and 4151 tumor cells in the low-PCD group (**Figure 3A**). The cell cycle states of all tumor cells in both groups were identified, with a higher proportion of tumor cells in the G2/M phase observed in the high-PCD group compared to the low-PCD group, indicating a more active proliferation of tumor cells in this group (**Figure 3B**).

Through differential analysis of the two groups of tumor cells, we observed a significant upregulation of metabolism-associated genes (e.g., *APOE*, *FABP1*) in the low-PCD group, whereas a diverse array of cancer-promoting genes (e.g., *S100A11*, *LDHA*) exhibited heightened expression levels in the high-PCD group (**Figure 3C**). The KEGG pathway enrichment analysis revealed that metabolic pathways, such as those associated with glycine, serine and threonine metabolism, fatty acid metabolism, and carbon metabolism, were predominant in the tumor cells of the low-PCD group (**Figure 3D**). Furthermore, we conducted further analysis on metabolism-related signatures. In concordance with the findings of KEGG pathway enrichment analysis, we observed a significant increase in metabolism-related signatures in tumor cells in low-PCD group versus high-PCDscore group (**Figures 3E, F**). Functionally, tumor cells in the high-PCD group exhibited significantly higher expression levels of stemness, proliferation, and metastatic signatures compared to those in the low-PCD group, suggesting a higher degree of malignancy (**Figure 3G**).

### Exhaustion of T cell function in the high-PCD group

**Figure 4:**
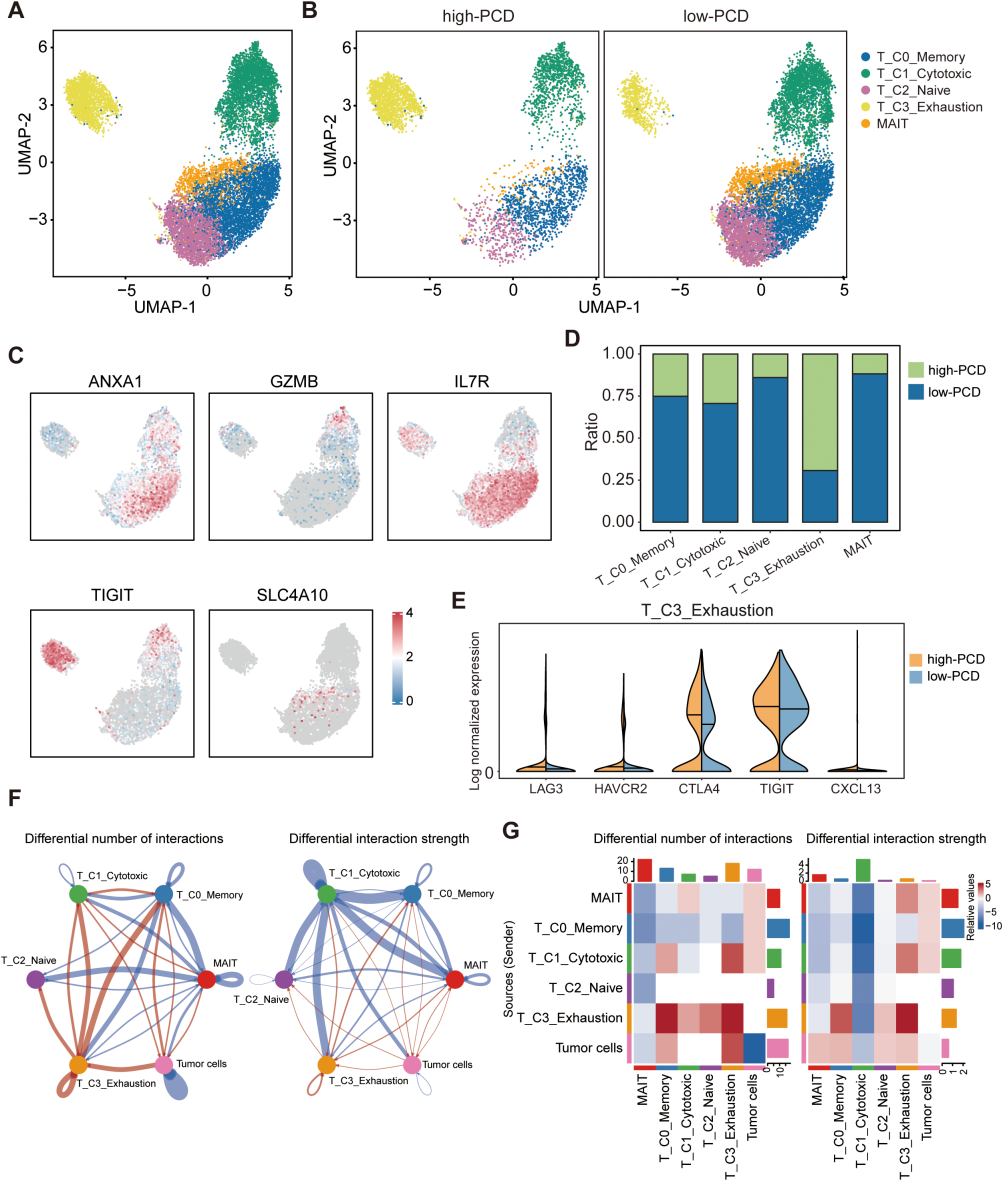
Landscape of tumor-infiltrating T cells in high- and low-PCD groups. **(A, B)** UMAP plot displaying five cell subtypes of T cells. **(C)** UMAP visualization plot depicting the expression patterns of selected marker genes for the defined T cell subtypes. **(D)** Bar plots illustrating the proportions of T cell subtypes in the high-PCD and low-PCD groups. **(E)** Violin plots depicting the distribution of expression levels for exhaustion-related genes. **(F)** The circle plots illustrate the differences in interaction number and strength among various cell populations. Red edges indicate increased signaling, while blue edges indicate decreased signaling in the high-PCD group compared to the low-PCD group. **(G)** The heatmaps illustrate differences in interaction number and strength among various cell populations. The top colored bar plot shows the sum of each column’s absolute values (incoming signaling), while the right colored bar plot shows the sum of each row’s absolute values (outgoing signaling). In the color bar, red represents increased signaling, whereas blue represents decreased signaling in the high-PCD group compared to the low-PCD group.

T cells were functionally annotated and reclustered into five distinct clusters, including T_C0_Memory, T_C1_Cytotoxic, T_C2_Naive, T_C3_Exhaustion, and MAIT (**Figures 4A, B**). The UMAP plots depict the expression levels of representative marker genes (*ANXA1*, *GZMB*, *IL7R*, *TIGIT*, *SLC4A10*) for each of the five defined T cell subpopulations mentioned above (**Figure 4C**). In the low-PCD group, there was an increase in the proportion of anti-tumor cells such as T_C1_Cytotoxic cells, whereas the high-PCD group showed an enrichment of T_C3_Exhaustion cells (**Figure 4D**). Further analysis demonstrated that exhaustion markers such as *LAG3*, *HAVCR2*, *CTLA4*, *TIGIT*, and *CXCL13* were relatively predominant in T cells from the high-PCD group (**Figure 4E**). These results suggest that the high-PCD group is more likely to exhibit an exhausted antitumor immune environment.

To investigate interactions exhibiting significant changes between cell populations, CellChat analyzes the number and strength of these interactions among different cell populations. Circle plots (**Figure 4F**) and heatmaps (**Figure 4G**) revealed that the number and strength of interactions in the cell-cell communication network between tumor cells and T_C3_Exhaustion cells were increased in the high-PCD group compared to the low-PCD group.

**Figure 5:**
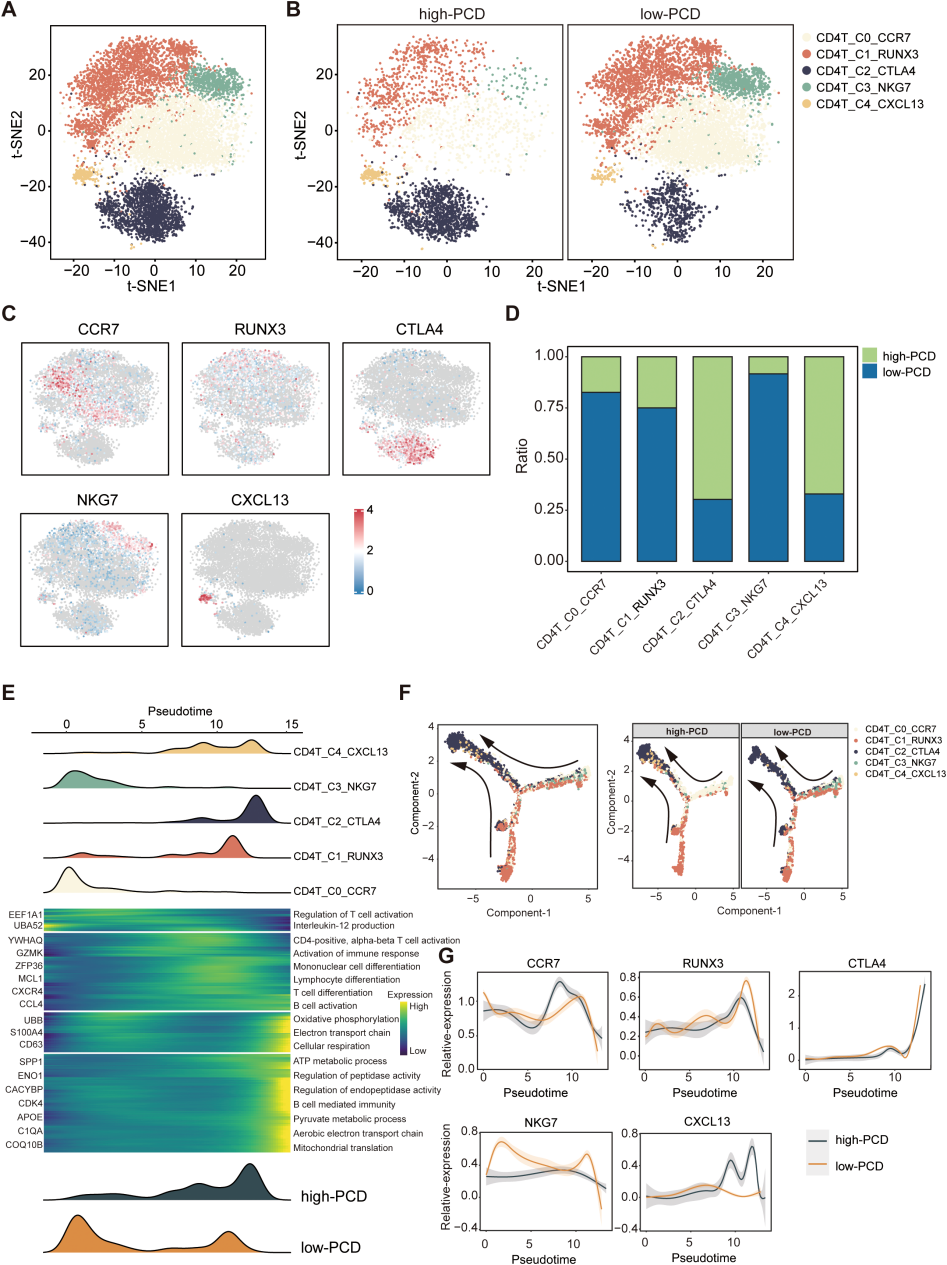
Characteristics of CD4+ T cells in high- and low-PCD groups. **(A, B)** t-SNE plot illustrating five cell subtypes of CD4+T cells. **(C)** t-SNE plot illustrating the expression patterns of selected marker genes for defined CD4+ T cell subtypes. **(D)** Bar plots depicting the proportions of CD4+ T cell subtypes in the two groups. **(E)** Heatmap illustrating the dynamic changes in gene expression of CD4+ T cells along the pseudotime. The upper panel displays the distribution of CD4+ T cell subsets, while the lower panel shows the distribution of total CD4+ T cells across the two groups along the pseudotime. **(F)** Pseudotime-ordered analysis of CD4+ T cells. **(G)** Two-dimensional plots displaying the expression of selected marker genes for defined CD4+ T cell subtypes along the pseudotime.

Next, T cells were further classified into CD4+T cell subtypes and CD8+T cell subtypes based on the expression levels of CD4 and CD8. By performing reclustering analyses of scRNA-seq data, we identified five subtypes of CD4+ T cells: CD4+T_C0_CCR7 (CD4+ naive T cells), CD4+T_C1_RUNX3 (CD4+ helper T cells), CD4+T_ C2_CTLA4 (CD4+ exhausted T cells), CD4+T_C3_NKG7 (CD4+ effector T cells), and CD4+T_C4_CXCL13 (CD4+ regulatory T cells) (**Figures 5A–C**). Importantly, the proportions of exhausted CD4+T_C2_CTLA4 cells and regulatory T cells (Tregs) CD4+T_C4_CXCL13 were significantly higher in the high-PCD group, while the proportion of effector CD4+T_C3_NKG7 cells was enriched in the low-PCD group (**Figure 5D**). Subsequently, we investigated dynamic immune states and cell transcriptional profiles in CD4+ T cells. The results indicated that CD4+ T cells exhibited similar transition trajectories in both high-PCD and low-PCD groups, albeit in distinct states. This transition was identified as progressing from CD4+ naive T cells (CD4+T_C0_CCR7) and CD4+ effector T cells (CD4+T_C3_NKG7), through intermediate transitional states characterized by CD4+ helper T cells (CD4+T_C1_RUNX3) and CD4+ regulatory T cells (CD4+T_C4_CXCL13), to an exhausted state characterized by CD4+ exhausted T cells (CD4+T_ C2_CTLA4) (**Figure 5E**). It is noteworthy that early-stage CD4+ T cells and terminally exhausted CD4+ T cells were primarily found in the low-PCD group, whereas CD4+ T cells in transitional and terminally exhausted states were predominantly observed in the high-PCD group (**Figures 5E, F**). Furthermore, marker gene trajectory analysis affirmed a heightened state of terminally exhausted CD4+ T cells in the high-PCD group (**Figure 5G**).

CD8+ T cells were also identified into 5 subtypes, namely CD8+T_C0_CCL5 (effector memory CD8+T cells), CD8+T_C1_GNLY (effector memory CD8+T cells), CD8+T_C2_IL7R (naive CD8+T cells), CD8+T_C3_GZMK (effector CD8+T cells), and CD8+T_C4_CTLA4 (exhausted CD8+T cells) (**Supplementary Figures S3A-C**). Similarly, the proportion of exhausted CD8+ T cells (CD8+T_C4_CTLA4) was significantly higher in the high-PCD group compared to the low-PCD group (**Supplementary Figure S3D**), and the exhaustion score of CD8+ cells in the high-PCD group was significantly higher (**Supplementary Figure S3E**).

### Profiling of tumor microenvironment in high- and low-PCD spots by ST-seq

**Figure 6:**
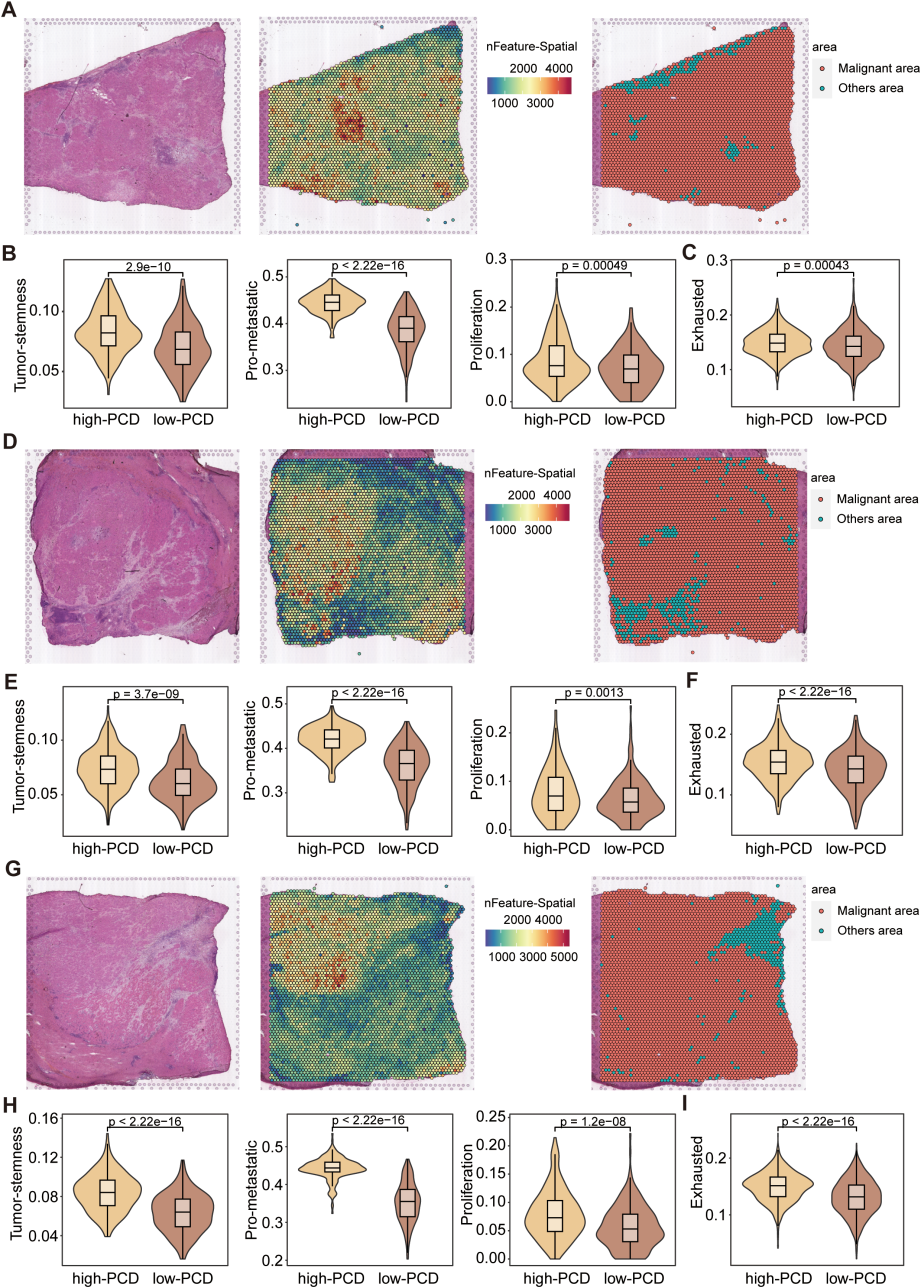
Spatial transcriptomics analysis of HCC samples. **(A, D, G)** Representative H&E-stained images of HCC tissues (left), spatial transcriptomic feature counts (middle), and classification of malignant and non-malignant areas (right) based on nFeature_Spatial counts. **(B, E, H)** Violin plots displaying the expression scores of stemness, proliferation, and metastatic signatures in high-PCD and low-PCD spots, with p-values from the Wilcoxon test. **(C, F, I)** Violin plots depicting the exhaustion signature scores in high-PCD and low-PCD spots, with p-values from the Wilcoxon test.

Similarly, the ST-seq analysis of three representative HCC samples revealed distinct differences in tumor microenvironment features between high-PCD and low-PCD spots. Representative hematoxylin and eosin (H&E) stainin and spatial feature plots indicate variations in spatial transcriptomic features distribution across malignant and non-malignant areas (**Figures 6A, D, G**). Functionally, high-PCD spots demonstrated significantly elevated expression levels of tumor stemness, proliferation, and metastatic signatures compared to low-PCD spots, indicating a greater degree of malignancy (**Figures 6B, E, H**). Compared to the spots surrounding low-PCD spots, the spots surrounding high-PCD spots exhibited significantly higher exhaustion scores, suggesting an immunosuppressive microenvironment around high-PCD spots (**Figures 6C, F, I**).

### Oncogenic role of the prognostic marker gene UBE2E1 in HCC

**Figure 7:**
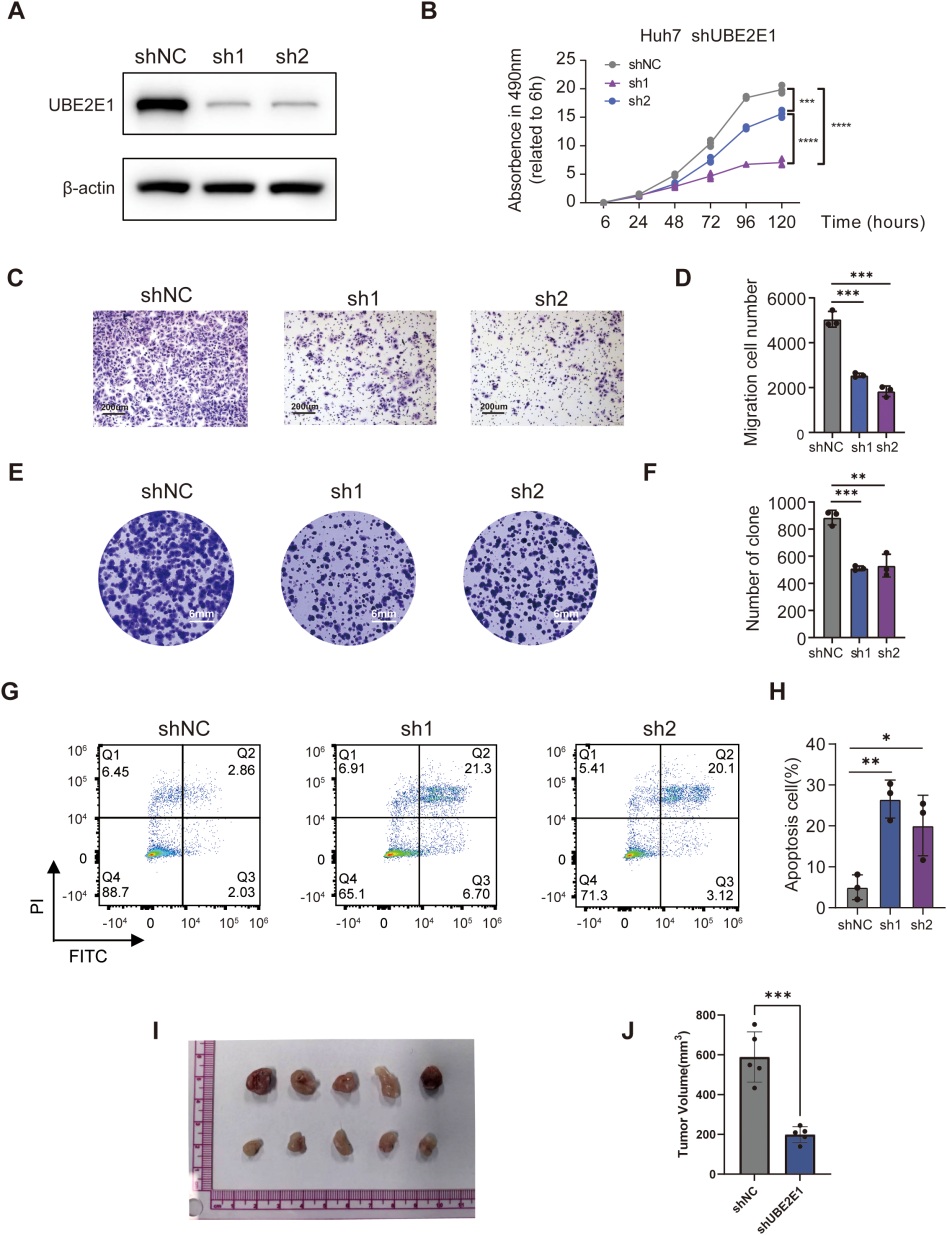
UBE2E1 depletion impedes HCC cells survival, proliferation and migration. **(A)** Western blotting confirmation of UBE2E1 depletion using two independent short hairpin RNAs (shRNAs) in Huh7 cells. **(B)** Cell Counting Kit-8 assay (CCK8) of UBE2E1-depleted and control Huh7. **(C, D)** Colony-formation assay of UBE2E1-depleted and control Huh7. Left panels: representative images with a 200 μm scale bar. Right panels: quantification data. **(E, F)** Transwell cell migration analysis of UBE2E1-depleted and control Huh7 cells. Left panels: representative images with a 6 mm scale bar. Right panels: quantification data. **(G, H)**Annexin V/PI analysis of UBE2E1-depleted and control Huh7 cells. Left panels: representative images. Right panels: quantification data. **(I, J)** Tumor growth analysis in UBE2E1-depleted and control Huh7 cells. Left panels: representative image of tumor xenografts from shNC and shUBE2E1 groups with a scale bar in cm. Right panels: tumor volume measurements for each group. Data are presented as mean ± SD. *p < 0.05, **p < 0.01, **** p < 0.001, ****p < 0.0001 (one-way ANOVA; Student’s t test). Sh1, shUBE2E1-1; sh2, shUBE2E1-2; shNC, negative control shRNA. All *in vitro* assays were biologically repeated three times.

To gain deeper insights into the underlying mechanisms of the PCD prognostic model, we further investigated the genes associated with the model. Among these, UBE2E1, which has been less studied, is highly expressed in tumors and significantly impacts prognosis (**Supplementary Figures S4A, B**). We subsequently explored the function of UBE2E1 in the progression of HCC. We employed two independent shRNAs to knock down UBE2E1 expression in the HCC cell line Huh7 Huh7 cells and SNU-449 cells (**Figure 7A**; **Supplementary Figure S4C**). Our data demonstrated that knocking down UBE2E1 in the Huh7 cell and SNU-449 cell lines inhibited cell growth (**Figure 7B**; **Supplementary Figure S4D**), colony formation (**Figures 7C, D**; **Supplementary Figures S4E, F**), and significantly reduced cell migration capabilities (**Figures 7E, F**; **Supplementary Figures S4G, H**). Furthermore, UBE2E1 knockdown markedly increased apoptosis in Huh7 cells and SNU-449 cells (**Figures 7G, H**; **Supplementary Figures S4I, J**). In addition to these *in vitro* findings, we further validated the functional role of UBE2E1 using a xenograft model. We injected Huh7 cells with UBE2E1 knockdown into the subcutaneous space of NCG mice and observed a significant inhibition of tumor growth *in vivo* (**Figures 7I, J**).

## Discussion

In this study, we developed and validated a prognostic gene signature based on PCD-related genes to predict OS in HCC patients. The high-PCD group consistently demonstrated poorer survival outcomes across both internal (TCGA_TestSet) and external (GSE14520) validation cohorts, underscoring the signature’s predictive accuracy and potential clinical utility. This could facilitate more accurate risk stratification and individualized treatment plans for HCC patients, aligning with the broader movement towards precision oncology. Furthermore, our findings underscore the importance of PCD mechanisms in HCC prognosis and reveal distinct cellular and molecular characteristics between high- and low-PCD groups.

PCD, a cell death mechanism regulated by diverse biomacromolecules, constitutes a key feature in tumorigenesis, potentially informing varied therapeutic strategies (34). In our study, we developed a signature comprising 17 PCD-related genes (ENO1, CDK4, RPS17, PDLIM1, KLHDC10, IGFBP3, UBE2E1, CBS, UBB, YWHAQ, SPP1, USF2, STAT6, PCK2, CACYBP, HDAC1, CD79A), which demonstrated predictive capability for OS in patients with HCC. ENO1, a glycolytic enzyme, plays crucial roles in multiple pathological processes, particularly cancer development (35). Its upregulation is common in HCC, especially in highly metastatic cells or tissues, and is closely associated with advanced tumor-node-metastasis (TNM) stage, low differentiation grade, and unfavorable prognosis in HCC patients (36). CDK4 stands for Cyclin-Dependent Kinase 4, which is a protein involved in cell cycle regulation. It forms complexes with cyclin D proteins, facilitating the transition from the G1 phase to the S phase of the cell cycle, promoting cell division. Dysregulation of CDK4, commonly observed in cancer, contributes to uncontrolled cell growth, making it a prime target for cancer treatment strategies, especially those focused on inhibiting cell proliferation (37).

The high-PCD group not only exhibited worse OS but also displayed distinctive tumor-intrinsic features and immune microenvironment characteristics. Tumor cells in this group showed heightened expression of signatures linked to cancer stemness, proliferation, and metastasis, suggesting a more aggressive tumor phenotype. This aligns with the increased prevalence of markers like S100A11 (38) and LDHA (39) in the high-PCD group, known for their roles in malignant progression. The KEGG pathway enrichment analysis highlighted significant metabolic activity in the low-PCD group, with pathways such as glycine, serine, and threonine metabolism being predominant. This metabolic profile is often associated with less aggressive tumors (40), potentially explaining the better survival outcomes observed in the low-PCD group. The metabolic reprogramming in the low-PCD tumors suggests a reliance on oxidative phosphorylation and other metabolic processes that may be less conducive to rapid tumor growth and metastasis (41). In contrast, the high-PCD group’s upregulation of genes involved in glycolysis and other proliferative pathways points to a Warburg-like metabolic phenotype, commonly associated with aggressive cancer behavior (42). This distinction underscores the potential of targeting metabolic pathways as part of a therapeutic strategy (42), particularly in high-PCD group HCC patients.

The exploration of the tumor immune microenvironment using scRNA-seq data revealed marked differences between high- and low-PCD groups. A striking finding was the differential distribution of T cell subtypes. While anti-tumor T cell subsets, such as T_C1_Cytotoxic cells, were enriched in the low-PCD group, the high-PCD group exhibited a significant accumulation of exhausted T cells, characterized by the expression of exhaustion markers such as LAG3, HAVCR2, CTLA4, TIGIT, and CXCL13. The high-PCD group’s immune landscape suggests an immune microenvironment where chronic antigen stimulation has driven T cells into a state of functional exhaustion, posing a key barrier to effective anti-tumor immunity and potentially explaining the poorer clinical outcomes observed in these patients. Targeting these exhaustion pathways with immune checkpoint inhibitors or other immunomodulatory therapies could potentially reinvigorate these T cells, restoring their ability to combat tumor cells (43, 44).

Our detailed analysis of CD4+ T cells further illuminated the immune landscape in HCC. The high-PCD group exhibited an increased proportion of terminally exhausted CD4+ T cells, indicating a state of immune exhaustion that compromises anti-tumor immunity (45). Conversely, the low-PCD group primarily harbored early-stage naive and effector CD4+ T cells, implying a potentially more robust anti-tumor response (46). Supporting these findings, in the transition trajectory path, a progression from naive and effector states through intermediate states to terminal exhaustion in CD4+ T cells. This progression was more pronounced in the high-PCD group, suggesting that these CD4+ T cells are more likely to undergo exhaustion (45, 46). This finding is significant as it highlights potential intervention points where therapeutic strategies could be employed to alter the course of CD4+ T cell exhaustion, potentially improving patient outcomes.

Similarly, CD8+ T cells were categorized into five subtypes, with the high-PCD group exhibiting a higher proportion of exhausted CD8+ T cells (CD8+T_C4_CTLA4) and higher exhaustion scores. This finding reinforces the notion that elevated PCD scores correlate with a more exhausted immune microenvironment, thereby impairing the overall anti-tumor immune response. The presence of multiple subtypes of CD8+ T cells, including effector memory, naive, effector, and exhausted T cells, provides a detailed picture of the functional states within the tumor microenvironment. The high proportion of exhausted CD8+ T cells in the high-PCD group highlights the potential for therapeutic interventions aimed at reversing exhaustion and restoring cytotoxic function (47). For instance, therapies combining checkpoint inhibitors with agents that promote T cell activation and proliferation might be particularly effective in these patients (48).

The ST-seq analysis reveals spatial heterogeneity in the tumor microenvironment, with high-PCD spots exhibiting higher stemness, proliferation, and metastatic signatures, along with higher exhaustion scores compared to low-PCD spots. This spatial variation in transcriptomic features underscores the complexity of the tumor microenvironment and the importance of considering spatial context in understanding tumor biology. The immunosuppressive microenvironment around high-PCD spots suggests potential strategies for spatially targeted therapies to overcome local immune evasion mechanisms.

UBE2E1, a member of the ubiquitin-conjugating enzyme E2 class involved in the sequential enzymatic cascade of protein ubiquitination (E1, E2, and E3) (49), emerged as a pivotal prognostic marker with oncogenic properties among the PCD-related genes. Functional validation revealed that UBE2E1 knockdown significantly suppressed HCC cell growth, colony formation, and migratory capacity, while concurrently inducing apoptosis. These findings underscore UBE2E1’s critical role in HCC progression and its potential as a therapeutic target, further supported by its elevated expression in tumors and strong association with poor prognosis in our study.

The robust predictive capability of the PCD-related gene signature for HCC patient prognosis has several clinical implications. First, it could serve as a valuable tool for stratifying patients and tailoring therapeutic interventions based on individual risk profiles. High-PCD patients, characterized by aggressive tumor biology and immune exhaustion, may benefit from combinatorial therapies aimed at reinvigorating the immune response, such as immune checkpoint inhibitors. Conversely, patients with low PCD scores might respond better to therapies targeting metabolic pathways, reflecting their distinct tumor biology. Our findings underscore the importance of further research into the mechanisms underlying PCD and its interplay with the immune microenvironment. Understanding how different PCD pathways contribute to immune exhaustion and tumor progression could unveil new therapeutic targets and strategies. Additionally, integrating PCD-related biomarkers into clinical practice could enhance the precision of existing prognostic models and inform more nuanced treatment decisions. Our findings also suggest avenues for integrating PCD-related insights with emerging therapeutic modalities. For instance, the observed differences in metabolic and immune profiles between high- and low-PCD groups could inform the development of combination therapies. Targeting metabolic vulnerabilities in low-PCD tumors while simultaneously addressing immune exhaustion in high-PCD tumors could enhance therapeutic efficacy. Furthermore, the dynamic nature of T cell states revealed by our single-cell analyses points to the potential of temporal modulation of the immune response. Therapies that sequentially target different stages of T cell activation and exhaustion could provide a more comprehensive approach to restoring anti-tumor immunity. This could involve initial strategies to boost T cell activation and proliferation, followed by interventions to prevent or reverse exhaustion.

## Conclusions

In summary, our study provides a comprehensive analysis of the role of PCD-related genes in predicting HCC prognosis and highlights the distinct tumor and immune characteristics associated with different PCD scores. The identified gene signature offers a powerful prognostic tool and opens new avenues for personalized treatment strategies in HCC, emphasizing the need for continued research into the molecular mechanisms of cell death and immune regulation in cancer. Integrating PCD-related biomarkers into clinical workflows could significantly enhance the precision of HCC prognostication and treatment. By leveraging scRNA-seq and ST-seq analysis, we have delineated the cellular composition of the tumor microenvironment and identified functional disparities in tumor and immune cells between high- and low-PCD patients. These insights pave the way for more targeted and effective therapeutic interventions, potentially improving outcomes for HCC patients.

## Limitations

Despite the promising results, there are several limitations to this study that should be addressed in future research. One limitation is the use of publicly available datasets, including TCGA and GEO, which may not fully represent the diversity of the general population. The inclusion of only a limited number of datasets, particularly in specific subgroups, could potentially limit the generalizability of our findings. Furthermore, differences in the data acquisition methods between the TCGA (RNA-seq) and GEO (microarray) platforms, though addressed by normalization and batch effect correction, could still influence the robustness of the model’s predictions across diverse populations.

Another limitation is the reliance on bioinformatics models, which may not completely reflect the complexity of tumor biology in clinical settings. While we have demonstrated the predictive value of the PCD-based scoring system in stratifying patients based on prognosis, the clinical implementation of this model faces several challenges. The integration of such models into routine clinical practice requires further validation in multi-center clinical trials and standardization across different healthcare settings. Additionally, clinical implementation requires practical considerations such as the cost-effectiveness of high-throughput techniques (e.g., scRNA-seq and spatial transcriptomics) and their accessibility in routine diagnostics.

Lastly, while we identified UBE2E1 as a key oncogenic marker, further research is needed to understand its precise role and how its inhibition may affect therapeutic outcomes. The validation of specific biomarkers and their mechanisms in the clinical context, including drug development targeting PCD-related pathways, remains a challenge.

## Data Availability

The data utilized in this study are publicly available in the TCGA (https://xenabrowser.net) and GEO database (https://www.ncbi.nlm.nih.gov/geo/). The spatial transcriptomics sequencing data were collected from HRA000437 (https://ngdc.cncb.ac.cn/gsa-human/browse/HRA000437). The data presented in the study are deposited in the Figshare repository, accession number 10.6084/m9.figshare.29552894. The dataset can be accessed at: https://figshare.com/s/7ed4e82530d75b3b7bb3.

## Supplementary material

Supplementary Figure 1 Development and validation of the PCD scores prediction model. **(A)** A plot of partial likelihood deviance versus log(λ) was generated using LASSO regression. **(B)** The coefficients of the selected features are depicted based on the lambda parameter.

Supplementary Figure 2 Single-cell RNA sequencing (ScRNA-seq) landscape of high-PCD and low-PCD HCC patients. **(A)** The Uniform Manifold Approximation and Projection (UMAP) plot represents six major cell types across all HCC samples. **(B)** Cells are colored by predicted ploidy status: red indicates aneuploid cells (predicted tumor cells), blue indicates diploid cells (primarily T/NK cells), and gray represents cells not included in CopyKAT analysis (e.g., other stromal or immune cells). Copy number was annotated using CopyKAT, which predicts aneuploid cells independent of prior tumor cell identification. **(C)** Dotplot illustrating the expression patterns of signature genes in the specified cell types. **(D)** Bar plots depicting the proportions of high-PCD and low-PCD groups in each cell type. Cell cycle heterogeneity **(E)** and the respective proportions of cells **(F)** within the high- and low-PCD groups.

Supplementary Figure 3 Landscape of CD8+ T cells in high- and low-PCD groups. **(A, B)** t-SNE plot displaying five cell subtypes of CD8+ T cells. **(C)** t-SNE plot illustrating the expression patterns of selected marker genes for defined CD8+ T cell subtypes. **(D)** Bar plots illustrating the proportions of CD8+ T cell subtypes in the high-PCD and low-PCD groups. **(E)** Violin plots depicting the exhaustion signature scores in CD8+ T cells from the high-PCD and low-PCD groups; p values from the Wilcoxon test.

Supplementary Figure 4 Functional validation of UBE2E1 knockdown in HCC. **(A)** The relative expression of UBE2E1 in HCC (red point) and peri-tumor or normal tissues (blue point). **(B)** The Kaplan-Meier survival curve of OS and UBE2E1 expression. **(C)** Western blotting confirmation of UBE2E1 depletion using two independent shRNAs in SNU-449 cells. **(D)** CCK8 of UBE2E1-depleted and control SNU-449 cells. **(E, F)** Colony-formation assay of UBE2E1-depleted and control Huh7. Left panels: representative images with a 200 μm scale bar. Right panels: quantification data. **(G, H)** Transwell cell migration analysis of UBE2E1-depleted and control Huh7 cells. Left panels: representative images with a 6 mm scale bar. Right panels: quantification data. **(I, J)** Annexin V/PI analysis of UBE2E1-depleted and control Huh7 cells. Left panels: representative images. Right panels: quantification data. Data are presented as mean ± SD. **p< 0.01. ***p< 0.001. ****p< 0.0001 (one-way ANOVA; Student’s t test). Sh1, shUBE2E1-1; sh2, shUBE2E1-2; shNC, negative control shRNA. All *in vitro* assays were biologically repeated three times.

Supplementary Table 1 Programmed cell death (PCD) signatures. A comprehensive list of 2159 genes associated with 15 distinct forms of programmed cell death, including anoikis, apoptosis-like morphology, autophagy, cuproptosis, entotic cell death, extrinsic apoptosis, ferroptosis, immunogenic cell death, intrinsic apoptosis, lysosome-dependent cell death, necroptosis, necrosis-like morphology, necrosis, parthanatos, and pyroptosis. Gene sets were curated from KEGG, MSigDB, peer-reviewed publications, and the GeneCards database.

Supplementary Table 2 Marker genes of identified cell types in scRNA-seq data. Marker genes of identified cell types in single-cell RNA sequencing (scRNA-seq) data. Key marker genes used to annotate major cell types identified in the scRNA-seq analysis of HCC tissues, including tumor cells, NK/T cells, myeloid cells, B cells, endothelial cells, and fibroblasts. Marker selection was based on established literature and validated cell type–specific gene expression patterns.

Supplementary Table 3 Risk Scores and Clinical Information for TCGA-HCC Training Set Patients, Stratified by PCD Scores. The risk scores (PCD scores), risk group assignments (high- or low-PCD), and relevant clinical information for each patient in the training set (TCGA-HCC cohort). Patients were dichotomized using the median PCD score (1.9013) as the cut-off value.

Supplementary Table 4 PCD Scores and Group Classifications for TCGA-HCC Internal Validation Set Patients. Detailed PCD scores and group classifications for patients in the internal validation set (TCGA-HCC internal test cohort). The median PCD score (1.9227) within this cohort was used as the cut-off to define high- and low-PCD groups.

Supplementary Table 5 PCD Scores and Risk Group Labels for GSE14520 External Validation Set Patients. PCD scores and risk group labels for patients in the external validation cohort (GSE14520 dataset). A cohort-specific median PCD score (1.9039) was applied to stratify patients into high- and low-PCD groups.
