# Supplementary figures and images for: Integrative spatial and single-cell transcriptomics elucidate programmed cell death-driven tumor microenvironment dynamics in hepatocellular carcinoma
Source: Front Immunol. 2025 Jul 16;16:1589563. doi: 10.3389/fimmu.2025.1589563 (PMC12308848; doi:10.3389/fimmu.2025.1589563)

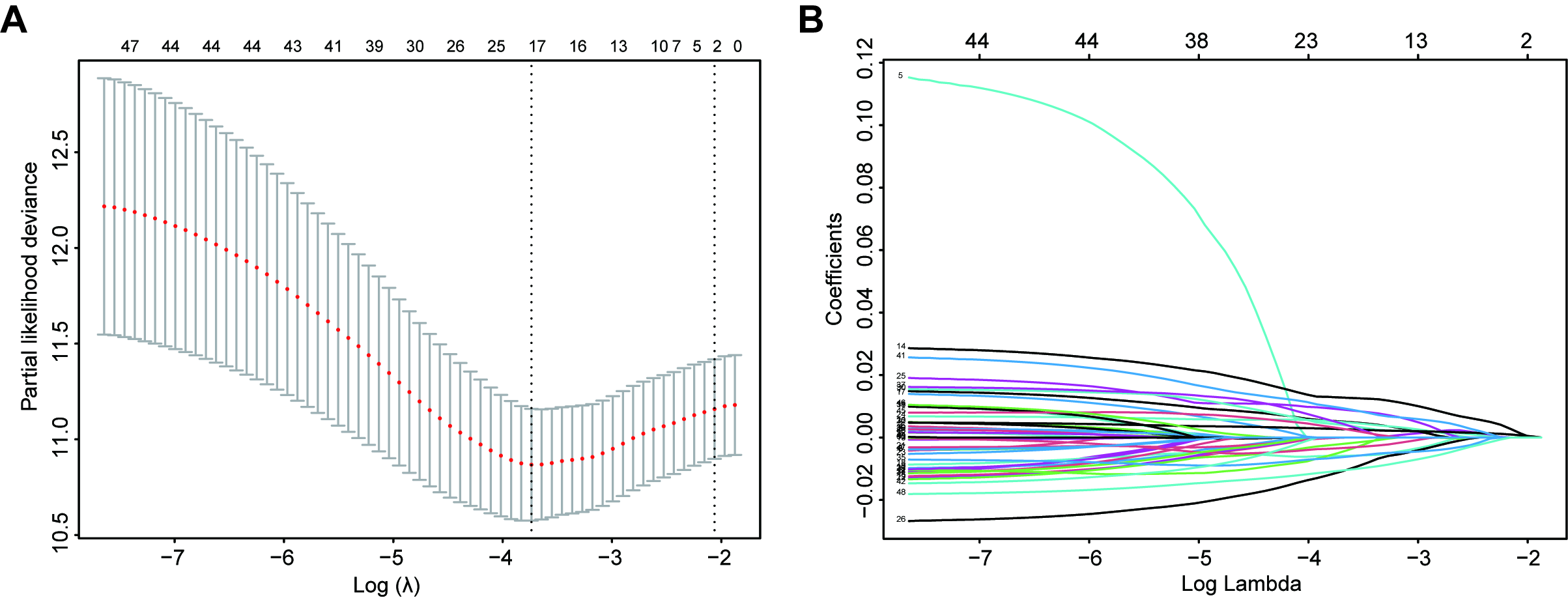

Supplement: Supplementary Figure 1 — Development and validation of the PCD scores prediction model. (A) A plot of partial likelihood deviance versus log(λ) was generated using LASSO regression. (B) The coefficients of the selected features are depicted based on the lambda parameter. [file Image1.tif]

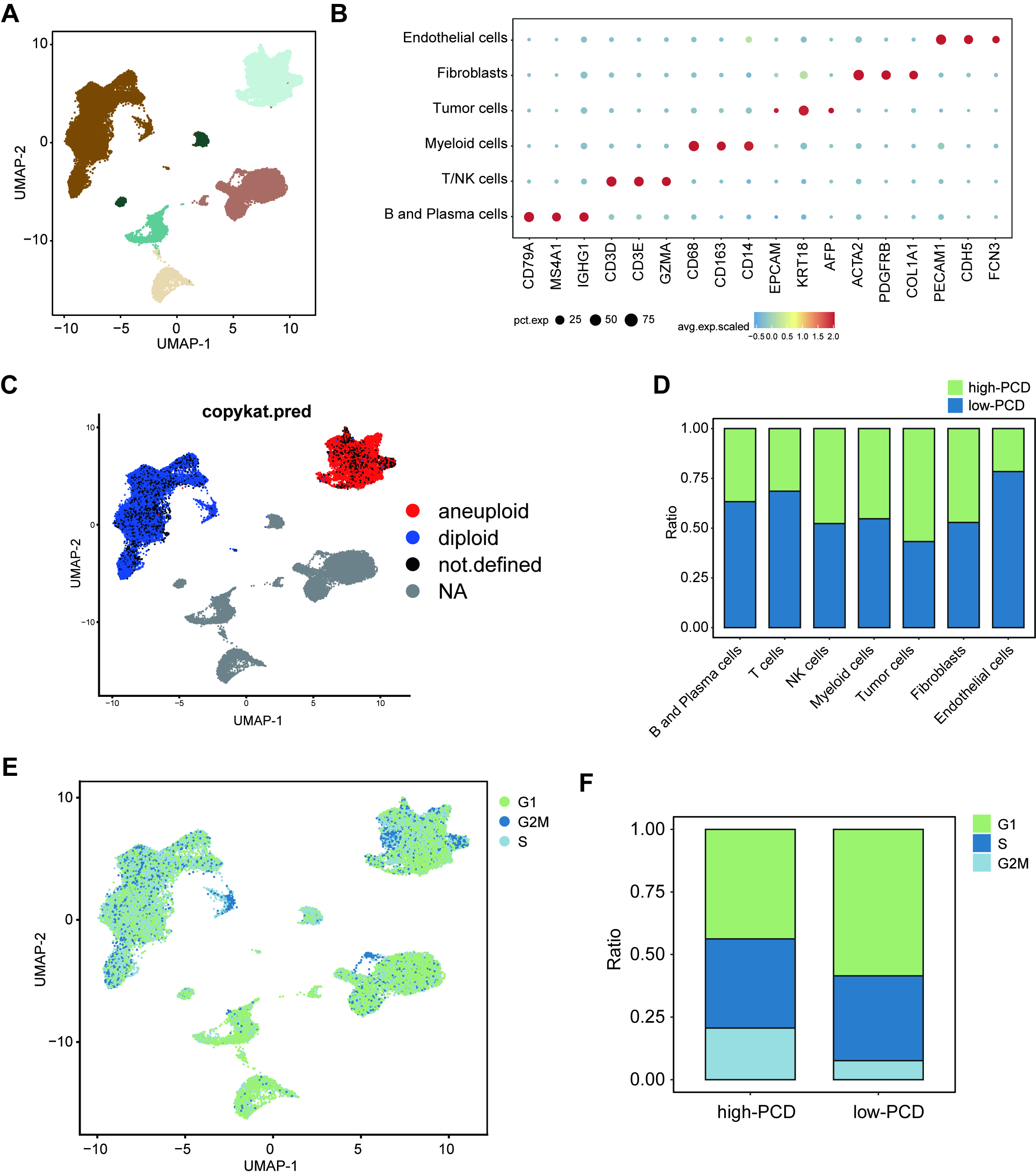

Supplement: Supplementary Figure 2 — Single-cell RNA sequencing (ScRNA-seq) landscape of high-PCD and low-PCD HCC patients. (A) The Uniform Manifold Approximation and Projection (UMAP) plot represents six major cell types across all HCC samples. (B) Cells are colored by predicted ploidy status: red indicates aneuploid cells (predicted tumor cells), blue indicates diploid cells (primarily T/NK cells), and gray represents cells not included in CopyKAT analysis (e.g., other stromal or immune cells). Copy number was annotated using CopyKAT, which predicts aneuploid cells independent of prior tumor cell identification. (C) Dotplot illustrating the expression patterns of signature genes in the specified cell types. (D) Bar plots depicting the proportions of high-PCD and low-PCD groups in each cell type. Cell cycle heterogeneity (E) and the respective proportions of cells (F) within the high- and low-PCD groups. [file Image2.tif]

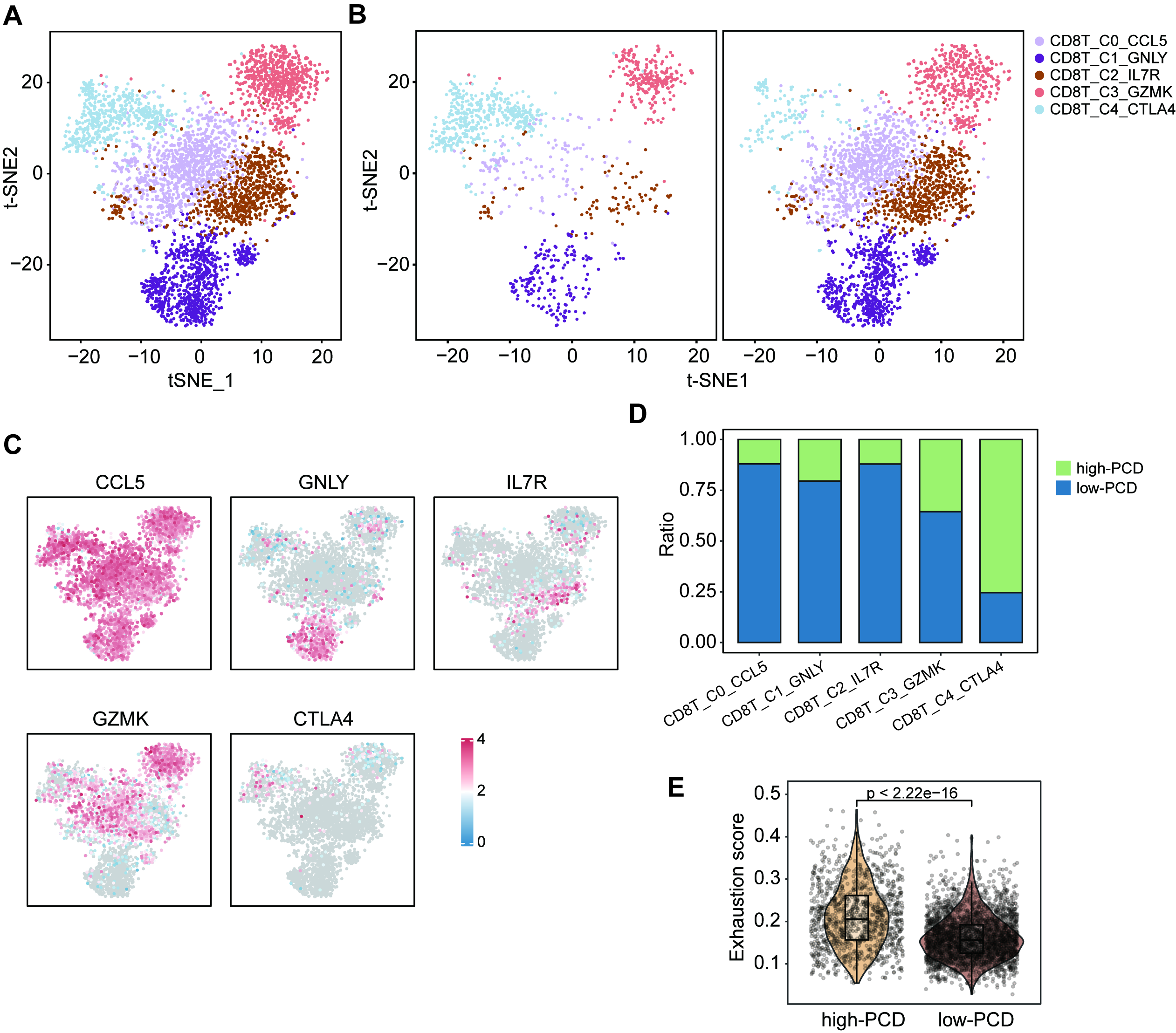

Supplement: Supplementary Figure 3 — Landscape of CD8+ T cells in high- and low-PCD groups. (A, B) t-SNE plot displaying five cell subtypes of CD8+ T cells. (C) t-SNE plot illustrating the expression patterns of selected marker genes for defined CD8+ T cell subtypes. (D) Bar plots illustrating the proportions of CD8+ T cell subtypes in the high-PCD and low-PCD groups. (E) Violin plots depicting the exhaustion signature scores in CD8+ T cells from the high-PCD and low-PCD groups; p values from the Wilcoxon test. [file Image3.tif]

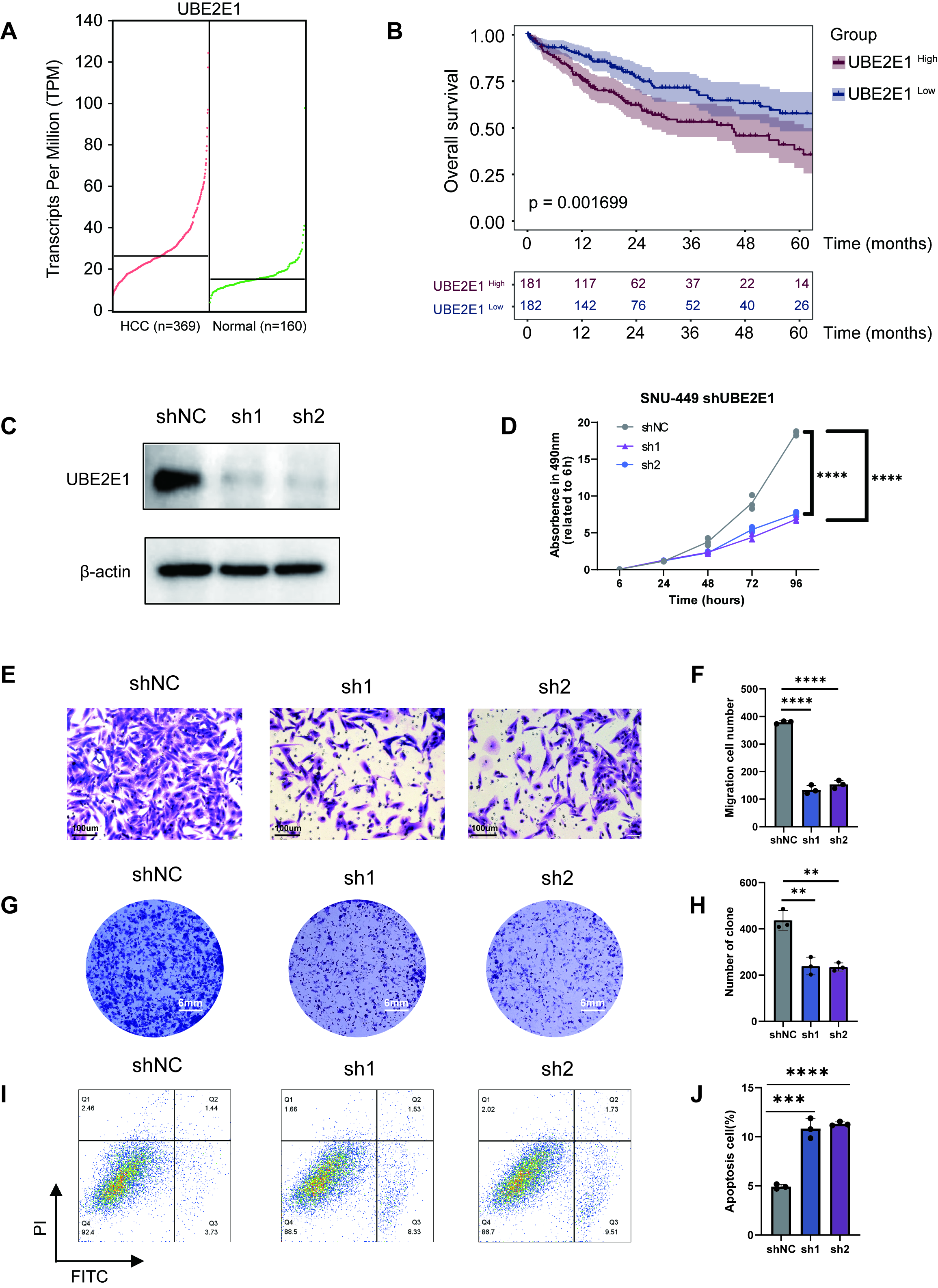

Supplement: Supplementary Figure 4 — Functional validation of UBE2E1 knockdown in HCC. (A) The relative expression of UBE2E1 in HCC (red point) and peri-tumor or normal tissues (blue point). (B) The Kaplan-Meier survival curve of OS and UBE2E1 expression. (C) Western blotting confirmation of UBE2E1 depletion using two independent shRNAs in SNU-449 cells. (D) CCK8 of UBE2E1-depleted and control SNU-449 cells. (E, F) Colony-formation assay of UBE2E1-depleted and control Huh7. Left panels: representative images with a 200 μm scale bar. Right panels: quantification data. (G, H) Transwell cell migration analysis of UBE2E1-depleted and control Huh7 cells. Left panels: representative images with a 6 mm scale bar. Right panels: quantification data. (I, J) Annexin V/PI analysis of UBE2E1-depleted and control Huh7 cells. Left panels: representative images. Right panels: quantification data. Data are presented as mean ± SD. **p< 0.01. ***p< 0.001. ****p< 0.0001 (one-way ANOVA; Student’s t test). Sh1, shUBE2E1-1; sh2, shUBE2E1-2; shNC, negative control shRNA. All in vitro assays were biologically repeated three times. [file Image4.tif]
