# Supplementary material for: Integrative spatial and single-cell transcriptomics elucidate programmed cell death-driven tumor microenvironment dynamics in hepatocellular carcinoma
Source: Front Immunol. 2025 Jul 16;16:1589563. doi: 10.3389/fimmu.2025.1589563 (PMC12308848; doi:10.3389/fimmu.2025.1589563)
Supplement: Supplementary Table 2 — Marker genes of identified cell types in scRNA-seq data. Marker genes of identified cell types in single-cell RNA sequencing (scRNA-seq) data. Key marker genes used to annotate major cell types identified in the scRNA-seq analysis of HCC tissues, including tumor cells, NK/T cells, myeloid cells, B cells, endothelial cells, and fibroblasts. Marker selection was based on established literature and validated cell type–specific gene expression patterns. [file Table2.docx]

**Supplemental Table S2. Marker genes of identified cell types in scRNA-seq data**

| **Cell types** | **Marker genes** | | |
| --- | --- | --- | --- |
| B and plasma cells | CD79A | MS4A1 | IGHG1 |
| Endothelial cells | PECAM1 | CDH5 | FCN3 |
| Tumor cells | EPCAM | KRT18 | AFP |
| Fibroblasts | ACTA2 | PDGFRB | COL1A1 |
| Myeloid cells | CD68 | CD163 | CD14 |
| T/NK cells | CD3D | CD3E | GZMA |
| T_C0_Memory | ANXA1 | CXCR4 | CCR6 |
| T_C1_Cytotoxic | GZMB | GZMH | GZMK |
| T_C2_Naive | CCR7 | LTB | IL7R |
| T_C3_Exhaustion | TIGIT | CTLA4 | BATF |
| MAIT | SLC4A10 | KLRB1 | NCR3 |
| CD4T_C0_CCR7 | CCR7 | LEF1 | TCF7 |
| CD4T_C1_RUNX3 | RUNX3 | CXCR4 | CCL5 |
| CD4T_C2_CTLA4 | CTLA4 | FOXP3 | LAYN |
| CD4T_C3_NKG7 | NKG7 | KLRG1 | CTSW |
| CD4T_C4_CXCL13 | CXCL13 | PDCD1 | BATF |
| CD8T_C0_CCL5 | CCL5 | GZMA | CD69 |
| CD8T_C1_GNLY | GNLY | KLRG1 | PRF1 |
| CD8T_C2_IL7R | IL7R | LTB | CCR7 |
| CD8T_C3_GZMK | GZMK | CXCR4 | CD44 |
| CD8T_C4_CTLA4 | CTLA4 | HAVCR2 | LAG3 |
