# Supplementary material for: Integrative spatial and single-cell transcriptomics elucidate programmed cell death-driven tumor microenvironment dynamics in hepatocellular carcinoma
Source: Front Immunol. 2025 Jul 16;16:1589563. doi: 10.3389/fimmu.2025.1589563 (PMC12308848; doi:10.3389/fimmu.2025.1589563)
Supplement: Supplementary Table 3 — Risk Scores and Clinical Information for TCGA-HCC Training Set Patients, Stratified by PCD Scores. The risk scores (PCD scores), risk group assignments (high- or low-PCD), and relevant clinical information for each patient in the training set (TCGA-HCC cohort). Patients were dichotomized using the median PCD score (1.9013) as the cut-off value. [file Table3.docx]

**Supplemental Table S3. Risk Scores and Clinical Information for TCGA-HCC Training Set Patients, Stratified by PCD Scores.**

| **id** | **riskScore** | **grouping** | |
| --- | --- | --- | --- |
| TCGA-DD-A4NG-01A  TCGA-G3-AAV4-01A  TCGA-BC-A10Y-01A  TCGA-K7-AAU7-01A  TCGA-BC-A10W-01A  TCGA-DD-AAD3-01A  TCGA-DD-AAC9-01A  TCGA-DD-AACT-01A  TCGA-GJ-A6C0-01A  TCGA-CC-5258-01A  TCGA-DD-AADP-01A  TCGA-ZS-A9CD-01A  TCGA-UB-A7MF-01A  TCGA-WX-AA46-01A  TCGA-CC-A8HV-01A  TCGA-DD-AADB-01A  TCGA-DD-A113-01A  TCGA-2Y-A9H9-01A  TCGA-XR-A8TD-01A  TCGA-CC-A7IF-01A  TCGA-DD-AACD-01A  TCGA-ED-A4XI-01A  TCGA-G3-A5SJ-01A  TCGA-DD-AAE2-01A  TCGA-EP-A2KC-01A  TCGA-ED-A7PX-01A  TCGA-DD-AADC-01A  TCGA-DD-AAVZ-01A  TCGA-CC-A3MA-01A  TCGA-ED-A627-01A  TCGA-2Y-A9GW-01A  TCGA-DD-AADK-01A  TCGA-DD-A1EH-01A  TCGA-G3-AAV7-01A  TCGA-DD-AACA-01A  TCGA-KR-A7K0-01A  TCGA-DD-AACC-01A  TCGA-UB-A7MD-01A  TCGA-DD-A4NS-01A  TCGA-DD-AAE3-01A  TCGA-DD-AACP-01A  TCGA-2Y-A9H8-01A  TCGA-FV-A3I1-01A  TCGA-2Y-A9H7-01A  TCGA-DD-AAVU-01A  TCGA-BC-A110-01A  TCGA-DD-AAE1-01A  TCGA-GJ-A3OU-01A  TCGA-RC-A7SB-01A  TCGA-CC-A8HS-01A  TCGA-FV-A3I0-01A  TCGA-2Y-A9GT-01A  TCGA-2Y-A9HA-01A  TCGA-FV-A2QQ-01A  TCGA-DD-AAEH-01A  TCGA-DD-AADM-01A  TCGA-G3-A3CK-01A  TCGA-EP-A26S-01A  TCGA-DD-A11A-01A  TCGA-XR-A8TE-01A  TCGA-DD-A73B-01A  TCGA-EP-A3JL-01A  TCGA-CC-A3MB-01A  TCGA-DD-AAD0-01A  TCGA-MI-A75I-01A  TCGA-DD-AACZ-01A  TCGA-CC-A8HU-01A  TCGA-RC-A6M5-01A  TCGA-2Y-A9GZ-01A  TCGA-LG-A6GG-01A  TCGA-DD-A4NV-01A  TCGA-G3-A25U-01A  TCGA-UB-AA0U-01A  TCGA-G3-A5SI-01A  TCGA-DD-A4ND-01A  TCGA-ED-A8O5-01A  TCGA-UB-A7ME-01A  TCGA-CC-5264-01A  TCGA-DD-AADA-01A  TCGA-2Y-A9HB-01A  TCGA-DD-AACS-01A  TCGA-DD-AAE0-01A  TCGA-G3-A5SK-01A  TCGA-BC-A69I-01A  TCGA-BC-A10R-01A  TCGA-BC-A10X-01A  TCGA-BC-A10S-01A  TCGA-DD-AAVQ-01A  TCGA-G3-A3CJ-01A  TCGA-BC-A10Q-01A  TCGA-CC-A1HT-01A  TCGA-DD-A11B-01A  TCGA-EP-A2KB-01A  TCGA-G3-A3CG-01A  TCGA-DD-AAVR-01A  TCGA-DD-AAVW-01A  TCGA-DD-A4NF-01A  TCGA-DD-AACN-01A  TCGA-DD-A3A2-01A  TCGA-K7-A5RF-01A  TCGA-BC-A10T-01A  TCGA-DD-AADJ-01A  TCGA-DD-AACE-01A  TCGA-DD-AADD-01A  TCGA-4R-AA8I-01A  TCGA-DD-AADS-01A  TCGA-G3-AAUZ-01A  TCGA-WX-AA47-01A  TCGA-NI-A4U2-01A  TCGA-DD-AACI-01A  TCGA-ZP-A9D1-01A  TCGA-UB-A7MB-01A  TCGA-DD-A4NR-01A  TCGA-RG-A7D4-01A  TCGA-5R-AAAM-01A  TCGA-BC-A217-01A  TCGA-UB-A7MC-01A  TCGA-MR-A8JO-01A  TCGA-DD-AAEG-01A  TCGA-DD-A39W-01A  TCGA-DD-A39Z-01A  TCGA-BC-A10Z-01A  TCGA-WQ-AB4B-01A  TCGA-CC-A123-01A  TCGA-YA-A8S7-01A  TCGA-2Y-A9H2-01A  TCGA-EP-A2KA-01A  TCGA-DD-AADW-01A  TCGA-DD-AAED-01A  TCGA-DD-AADV-01A  TCGA-ZS-A9CG-01A  TCGA-DD-AAW3-01A  TCGA-ZP-A9D2-01A  TCGA-G3-A25Y-01A  TCGA-K7-A5RG-01A  TCGA-EP-A3RK-01A  TCGA-CC-A7IJ-01A  TCGA-G3-AAV5-01A  TCGA-DD-A1ED-01A  TCGA-ED-A459-01A  TCGA-DD-AACL-01A  TCGA-MR-A520-01A  TCGA-DD-A1EF-01A  TCGA-G3-A25T-01A  TCGA-WX-AA44-01A  TCGA-DD-A4NE-01A  TCGA-CC-A7IG-01A  TCGA-ZP-A9D0-01A  TCGA-O8-A75V-01A  TCGA-DD-AADI-01A  TCGA-BC-A8YO-01A  TCGA-DD-A39V-01A  TCGA-DD-A1EC-01A  TCGA-DD-AACQ-01A  TCGA-DD-A1EB-01A  TCGA-KR-A7K7-01A  TCGA-DD-AADN-01A  TCGA-LG-A9QC-01A  TCGA-RC-A7S9-01A  TCGA-BC-A112-01A  TCGA-CC-A7IL-01A  TCGA-KR-A7K2-01A  TCGA-DD-AADU-01A  TCGA-ED-A66Y-01A  TCGA-CC-A8HT-01A  TCGA-BC-A216-01A  TCGA-RC-A7SK-01A  TCGA-DD-AAD8-01A  TCGA-DD-A119-01A  TCGA-G3-AAV6-01A  TCGA-DD-AAEK-01A  TCGA-DD-A11D-01A  TCGA-DD-A1EG-01A  TCGA-PD-A5DF-01A  TCGA-DD-A3A9-01A  TCGA-2Y-A9H4-01A  TCGA-DD-A4NL-01A  TCGA-5C-A9VH-01A  TCGA-3K-AAZ8-01A  TCGA-MI-A75G-01A  TCGA-DD-A3A6-01A  TCGA-CC-A5UD-01A  TCGA-DD-A3A3-01A  TCGA-CC-A7IH-01A  TCGA-BW-A5NO-01A  TCGA-XR-A8TG-01A  TCGA-CC-5259-01A  TCGA-2Y-A9H3-01A  TCGA-UB-AA0V-01A  TCGA-GJ-A9DB-01A  TCGA-DD-A4NK-01A  TCGA-2Y-A9H6-01A  TCGA-DD-AAD5-01A  TCGA-BD-A3EP-01A  TCGA-CC-5260-01A  TCGA-FV-A2QR-01A  TCGA-DD-AA3A-01A  TCGA-FV-A495-01A  TCGA-ZS-A9CE-01A  TCGA-5R-AA1D-01A  TCGA-MI-A75E-01A  TCGA-DD-AAEA-01A  TCGA-G3-A25V-01A  TCGA-CC-A9FS-01A  TCGA-BC-A69H-01A  TCGA-DD-A3A7-01A  TCGA-CC-5261-01A  TCGA-G3-AAV0-01A  TCGA-DD-AAE4-01A  TCGA-DD-A3A8-01A  TCGA-G3-A7M5-01A  TCGA-CC-A3MC-01A  TCGA-ED-A66X-01A  TCGA-G3-A6UC-01A  TCGA-BD-A3ER-01A  TCGA-DD-A3A5-01A  TCGA-DD-AACH-01A  TCGA-CC-5262-01A  TCGA-RC-A6M6-01A  TCGA-G3-A7M7-01A  TCGA-ES-A2HT-01A  TCGA-DD-AACX-01A  TCGA-2Y-A9GV-01A  TCGA-DD-AACF-01A  TCGA-UB-A7MA-01A  TCGA-G3-A25Z-01A  TCGA-MI-A75C-01A  TCGA-DD-A4NA-01A  TCGA-ZP-A9CV-01A  TCGA-T1-A6J8-01A  TCGA-DD-A1EK-01A  TCGA-5C-AAPD-01A  TCGA-KR-A7K8-01A  TCGA-DD-AAD6-01A  TCGA-DD-A11C-01A  TCGA-WJ-A86L-01A  TCGA-DD-AAEB-01A  TCGA-2Y-A9GU-01A  TCGA-DD-A73A-01A  TCGA-RC-A6M4-01A  TCGA-DD-AAVY-01A  TCGA-DD-A1EL-01A  TCGA-DD-A4NJ-01A  TCGA-DD-AACG-01A  TCGA-DD-AAC8-01A  TCGA-ED-A7XO-01A  TCGA-G3-A7M6-01A  TCGA-ED-A97K-01A  TCGA-DD-A73C-01A  TCGA-XR-A8TF-01A  TCGA-DD-A39Y-01A  TCGA-DD-A114-01A  TCGA-DD-AAEI-01A  TCGA-2Y-A9H5-01A | 2.356394657  2.54408114  2.086467892  2.479308452  3.30215398  1.234854441  1.344603123  1.188606164  3.059281577  2.921665498  1.69860525  2.210634587  2.324361501  1.361009471  2.316992785  1.827833861  2.286915454  1.276054412  1.505727003  1.382634854  1.602139625  1.717712173  1.900568361  1.499591984  1.785977934  2.645066112  2.471666945  1.657179339  2.602451252  1.266227532  2.074576587  1.173806674  2.226145355  3.476638315  1.514159297  2.098055939  1.00406954  2.248512044  1.699999643  1.318852328  2.437321852  2.292590167  2.057849144  2.323002544  1.725791373  1.694266238  2.013505303  1.845400764  1.296808425  2.032208402  2.336697716  1.153337678  2.402370021  1.997781137  0.837522787  2.264392363  1.527147208  1.805853645  2.880272632  2.242040161  3.033478261  2.042962269  2.264478877  2.602635895  2.624011685  2.754193992  2.82382147  1.880360155  1.190142684  2.58518943  0.981520219  1.416624726  2.006985256  2.622011967  1.883522828  1.262390297  1.114835637  3.002074679  0.570138697  1.623289309  2.016006228  2.28306318  1.583478464  1.973898225  2.52587115  2.059263219  1.402078438  0.793106564  1.554287234  2.605574096  2.476450361  1.365653054  1.985047111  1.990504806  0.966429075  1.147582566  2.235562382  1.493400473  1.142506685  1.219292081  2.380079714  2.229577928  1.430527284  1.902072601  1.800198453  0.43282  1.007437293  2.087339493  1.537818564  1.815947267  1.907419552  2.252394852  2.233348408  1.188993305  1.98560028  1.751798865  1.561238083  2.59365424  1.781356949  2.264899888  1.568905967  2.085919026  1.385295615  1.815660054  2.806116882  1.878705383  2.278975899  2.606668842  1.228054924  1.746549601  2.166558744  1.534709593  2.469089488  2.523242071  1.124115914  2.222836338  2.999252433  2.520566951  0.974665323  2.710265746  2.504697714  1.507719915  1.934338165  2.28674994  2.134520694  2.336658978  3.102839362  1.726998665  1.977149978  1.481039673  2.760593843  2.318614113  1.437952516  1.505247764  1.369477182  1.986082922  1.53444758  1.362878725  1.588051335  2.76797717  1.75387346  1.313496714  1.690672615  2.236106419  2.672771573  1.719812989  1.014222939  1.56183046  2.558913949  2.538655522  0.316094655  1.29388471  2.018217393  2.48521232  1.784261096  1.67420214  1.147345424  1.957783066  1.46637974  1.22129004  0.788754475  2.90258158  2.307774777  1.572909909  1.794575423  2.119310066  2.026322067  0.831461351  2.136533349  1.665594941  1.157144905  0.9926335  2.339052883  0.855896803  2.238576284  2.325524847  2.561387311  1.646712818  1.346281754  2.508591657  1.71973475  1.33050097  2.091073084  1.69214172  2.942390216  2.401332375  1.430223758  1.03004913  1.2680724  1.845635193  1.036665642  2.386715058  1.771963639  0.923438237  2.392356741  1.7722913  2.369178146  2.817509827  2.571145483  1.816895012  2.147036508  2.199154384  1.287233523  2.271347414  2.011087633  2.084918189  2.381026598  1.514975455  1.821087403  2.300014168  2.821375905  1.308666426  1.275913284  2.160707752  1.989946038  1.287158695  0.905103917  1.555006581  1.21772449  1.800816522  1.806471127  2.951342376  2.166769252  2.09791544  2.720984413  1.4286918  2.011365664  1.553436364  0.933748088  1.637979604  3.650389277  2.392129377  2.109626994  1.737361607 | | high  high  high  high  high  low  low  low  high  high  low  high  high  low  high  low  high  low  low  low  low  low  low  low  low  high  high  low  high  low  high  low  high  high  low  high  low  high  low  low  high  high  high  high  low  low  high  low  low  high  high  low  high  high  low  high  low  low  high  high  high  high  high  high  high  high  high  low  low  high  low  low  high  high  low  low  low  high  low  low  high  high  low  high  high  high  low  low  low  high  high  low  high  high  low  low  high  low  low  low  high  high  low  low  low  low  low  high  low  low  low  high  high  low  high  low  low  high  low  high  low  high  low  low  high  low  high  high  low  low  high  low  high  high  low  high  high  high  low  high  high  low  high  high  high  high  high  low  high  low  high  high  low  low  low  high  low  low  low  high  low  low  low  high  high  low  low  low  high  high  low  low  high  high  low  low  low  high  low  low  low  high  high  low  low  high  high  low  high  low  low  low  high  low  high  high  high  low  low  high  low  low  high  low  high  high  low  low  low  low  low  high  low  low  high  low  high  high  high  low  high  high  low  high  high  high  high  low  low  high  high  low  low  high  high  low  low  low  low  low  low  high  high  high  high  low  high  low  low  low  high  high  high  low |
