# Supplementary material for: Integrative spatial and single-cell transcriptomics elucidate programmed cell death-driven tumor microenvironment dynamics in hepatocellular carcinoma
Source: Front Immunol. 2025 Jul 16;16:1589563. doi: 10.3389/fimmu.2025.1589563 (PMC12308848; doi:10.3389/fimmu.2025.1589563)
Supplement: Supplementary Table 4 — PCD Scores and Group Classifications for TCGA-HCC Internal Validation Set Patients. Detailed PCD scores and group classifications for patients in the internal validation set (TCGA-HCC internal test cohort). The median PCD score (1.9227) within this cohort was used as the cut-off to define high- and low-PCD groups. [file Table4.docx]

**Supplemental Table S4. PCD Scores and Group Classifications for TCGA-HCC Internal Validation Set Patients.**

| **id** | **riskScore** | **grouping** | |
| --- | --- | --- | --- |
| TCGA-2Y-A9H1-01A  TCGA-DD-AACV-01A  TCGA-DD-A1EI-01A  TCGA-DD-AACW-01A  TCGA-DD-AADQ-01A  TCGA-DD-A1EA-01A  TCGA-5R-AA1C-01A  TCGA-CC-A7IE-01A  TCGA-DD-AAD2-01A  TCGA-DD-AACY-01A  TCGA-CC-A9FW-01A  TCGA-G3-A7M9-01A  TCGA-2Y-A9GS-01A  TCGA-2Y-A9GY-01A  TCGA-DD-AADO-01A  TCGA-FV-A3R3-01A  TCGA-BC-A3KG-01A  TCGA-G3-AAV2-01A  TCGA-CC-A7IK-01A  TCGA-G3-A25S-01A  TCGA-DD-AADR-01A  TCGA-ZP-A9CZ-01A  TCGA-DD-AAVS-01A  TCGA-DD-AAE6-01A  TCGA-DD-AACO-01A  TCGA-ED-A7PZ-01A  TCGA-DD-A73G-01A  TCGA-G3-AAV3-01A  TCGA-BC-A5W4-01A  TCGA-EP-A12J-01A  TCGA-FV-A4ZQ-01A  TCGA-DD-AAVX-01A  TCGA-RC-A7SH-01A  TCGA-DD-A115-01A  TCGA-DD-A1EE-01A  TCGA-ZP-A9CY-01A  TCGA-FV-A23B-01A  TCGA-HP-A5MZ-01A  TCGA-DD-AACK-01A  TCGA-ZP-A9D4-01A  TCGA-HP-A5N0-01A  TCGA-DD-A4NN-01A  TCGA-ES-A2HS-01A  TCGA-DD-AAD1-01A  TCGA-DD-A73D-01A  TCGA-DD-A116-01A  TCGA-DD-A4NI-01A  TCGA-FV-A3R2-01A  TCGA-DD-AAE7-01A  TCGA-FV-A496-01A  TCGA-DD-A39X-01A  TCGA-DD-AAE9-01A  TCGA-DD-AAVP-01A  TCGA-DD-A3A4-01A  TCGA-DD-A4NQ-01A  TCGA-5C-A9VG-01A  TCGA-CC-A3M9-01A  TCGA-LG-A9QD-01A  TCGA-G3-A3CH-01A  TCGA-G3-AAV1-01A  TCGA-ED-A7PY-01A  TCGA-G3-A3CI-01A  TCGA-MI-A75H-01A  TCGA-ED-A7XP-01A  TCGA-ED-A5KG-01A  TCGA-2Y-A9GX-01A  TCGA-DD-A73E-01A  TCGA-QA-A7B7-01A  TCGA-FV-A4ZP-01A  TCGA-G3-A25X-01A  TCGA-XR-A8TC-01A  TCGA-BC-A3KF-01A  TCGA-DD-AAVV-01A  TCGA-2Y-A9H0-01A  TCGA-DD-AADY-01A  TCGA-RC-A7SF-01A  TCGA-DD-AAEE-01A  TCGA-DD-A73F-01A  TCGA-DD-A4NP-01A  TCGA-DD-A118-01A  TCGA-DD-A4NH-01A  TCGA-K7-A6G5-01A  TCGA-DD-AACU-01A  TCGA-DD-A1EJ-01A  TCGA-BD-A2L6-01A  TCGA-G3-A5SM-01A  TCGA-WQ-A9G7-01A  TCGA-DD-AAW2-01A  TCGA-CC-5263-01A  TCGA-BC-A10U-01A  TCGA-G3-A7M8-01A  TCGA-ED-A82E-01A  TCGA-DD-A3A1-01A  TCGA-ZS-A9CF-01A  TCGA-CC-A7II-01A  TCGA-DD-AADG-01A  TCGA-DD-AAW0-01A  TCGA-DD-AACJ-01A  TCGA-DD-AADL-01A  TCGA-DD-A4NO-01A  TCGA-CC-A5UE-01A  TCGA-DD-A4NB-01A  TCGA-ED-A8O6-01A  TCGA-G3-A5SL-01A  TCGA-DD-AACB-01A  TCGA-DD-AAW1-01A  TCGA-CC-A5UC-01A  TCGA-NI-A8LF-01A  TCGA-DD-AADF-01A | 1.715748148  1.773553093  2.301935153  1.711141913  1.888426459  1.149788669  2.985265421  2.221692877  1.922684122  2.190966663  2.530018425  2.89607011  2.088240405  1.970038201  1.684284231  1.79565419  2.947317203  1.96151545  2.927112068  2.431654059  2.699424219  1.585777266  1.868140256  1.561786674  1.26384251  2.409344626  1.995912952  1.229328271  2.623759072  1.442765035  1.075470611  1.864761703  1.094705073  2.013112511  2.386634108  1.616151148  1.983797144  1.886712209  2.346977118  1.219286598  1.379392099  2.357251306  1.054233416  1.129590816  1.67755831  1.791315165  1.244056454  2.59854787  1.259561426  2.288625628  1.967274608  2.00228927  1.969188627  2.312093533  3.272936338  1.918591529  2.659911548  0.828986075  0.95577337  2.569050018  1.490901156  1.07513927  1.97722583  1.913816517  0.854292159  1.64642452  2.159203895  2.462575896  2.333446747  3.246017507  1.56123504  1.542427403  1.849234479  2.822386676  1.400295513  1.326566699  2.214299489  1.531002879  1.166321962  2.255598598  3.112099632  2.014075485  1.617431233  2.854953439  1.837532879  1.392041158  1.779256405  1.475711813  3.274510305  2.50800945  1.414665814  1.968833267  2.919474506  2.121996562  2.500291463  1.603207882  1.445092955  2.200883356  1.924473779  1.946502361  2.755512228  2.138107383  2.276596892  1.704610535  1.787273168  1.583821037  2.876952326  1.676691502  1.222342537 | | low  low  high  low  low  low  high  high  high  high  high  high  high  high  low  low  high  high  high  high  high  low  low  low  low  high  high  low  high  low  low  low  low  high  high  low  high  low  high  low  low  high  low  low  low  low  low  high  low  high  high  high  high  high  high  high  high  low  low  high  low  low  high  low  low  low  high  high  high  high  low  low  low  high  low  low  high  low  low  high  high  high  low  high  low  low  low  low  high  high  low  high  high  high  high  low  low  high  high  high  high  high  high  low  low  low  high  low  low |
