# Supplementary material for: Integrative spatial and single-cell transcriptomics elucidate programmed cell death-driven tumor microenvironment dynamics in hepatocellular carcinoma
Source: Front Immunol. 2025 Jul 16;16:1589563. doi: 10.3389/fimmu.2025.1589563 (PMC12308848; doi:10.3389/fimmu.2025.1589563)
Supplement: Supplementary Table 5 — PCD Scores and Risk Group Labels for GSE14520 External Validation Set Patients. PCD scores and risk group labels for patients in the external validation cohort (GSE14520 dataset). A cohort-specific median PCD score (1.9039) was applied to stratify patients into high- and low-PCD groups. [file Table5.docx]

**Supplemental Table S5. PCD Scores and Risk Group Labels for GSE14520 External Validation Set Patients.**

| **id** | **riskScore** | **grouping** | |
| --- | --- | --- | --- |
| GSM363205  GSM363115  GSM362970  GSM363354  GSM363039  GSM363209  GSM363344  GSM363271  GSM363404  GSM363217  GSM363053  GSM363295  GSM363272  GSM363164  GSM362965  GSM363126  GSM363082  GSM363176  GSM363099  GSM363316  GSM363178  GSM363343  GSM363033  GSM363055  GSM363100  GSM363249  GSM363130  GSM363149  GSM363337  GSM363031  GSM363166  GSM363049  GSM363032  GSM363072  GSM363289  GSM363296  GSM363235  GSM362958  GSM363366  GSM363311  GSM363071  GSM363329  GSM363200  GSM362984  GSM363037  GSM363362  GSM363357  GSM363080  GSM363125  GSM363106  GSM362988  GSM363107  GSM363355  GSM363087  GSM363268  GSM363015  GSM363014  GSM363364  GSM363034  GSM363078  GSM363202  GSM363332  GSM363313  GSM363017  GSM363294  GSM363330  GSM363360  GSM363008  GSM363143  GSM363127  GSM363102  GSM363151  GSM363124  GSM712542  GSM363335  GSM363350  GSM363386  GSM363207  GSM362986  GSM362972  GSM363186  GSM363288  GSM363016  GSM363341  GSM363243  GSM363168  GSM363051  GSM363098  GSM363314  GSM363170  GSM363029  GSM363298  GSM363265  GSM363038  GSM363309  GSM363104  GSM363393  GSM363011  GSM363180  GSM363048  GSM363077  GSM363144  GSM363336  GSM363073  GSM362977  GSM363012  GSM363328  GSM363215  GSM363400  GSM363358  GSM363391  GSM363333  GSM363083  GSM363218  GSM363086  GSM363267  GSM362994  GSM363224  GSM363269  GSM363297  GSM363182  GSM363109  GSM363054  GSM363075  GSM363129  GSM363101  GSM363378  GSM363232  GSM362993  GSM363150  GSM363142  GSM363013  GSM363331  GSM363128  GSM363315  GSM363384  GSM363147  GSM363368  GSM363081  GSM363052  GSM362966  GSM363169  GSM363085  GSM363388  GSM363196  GSM363266  GSM363326  GSM363230  GSM363237  GSM363204  GSM362992  GSM363264  GSM363312  GSM363123  GSM363245  GSM363192  GSM363291  GSM363273  GSM363148  GSM363263  GSM362978  GSM363056  GSM363310  GSM363275  GSM363352  GSM363293  GSM363292  GSM363211  GSM363070  GSM363339  GSM363270  GSM363371  GSM363009  GSM362982  GSM363188  GSM363251  GSM363057  GSM363030  GSM363274  GSM363146  GSM362983  GSM363222  GSM363074  GSM363290  GSM362987  GSM363035  GSM363036  GSM363079  GSM363317  GSM363172  GSM363084  GSM363239  GSM363121  GSM363108  GSM363152  GSM363105  GSM363327  GSM363194  GSM363346  GSM363174  GSM362976  GSM363190  GSM363069  GSM363247  GSM363076  GSM363376  GSM362959  GSM363226  GSM363010  GSM363220  GSM362964  GSM363348  GSM363213  GSM363198  GSM363184  GSM362960  GSM363145  GSM363050  GSM363241  GSM363122  GSM362971 | 1.219610261  1.33064495  1.421352889  2.901077956  1.135120334  1.617382998  1.741220226  2.674924137  1.473902216  1.653268064  2.156681545  0.804374473  1.481920789  1.395779327  1.851980674  2.702614306  1.308081459  1.360663851  1.943448469  1.857687409  1.827037844  1.11314422  2.207882892  1.525772154  3.306643643  1.278908993  1.007070551  1.350164833  2.300943971  2.122978294  2.408676294  2.42120008  2.353374218  1.709141906  1.29846259  2.528617273  2.261053782  1.892776941  0.751689834  1.853075266  2.170135601  1.953825179  1.783779522  0.482766891  2.401735015  2.044608535  2.223717136  2.646892608  2.04149512  2.051010013  1.705091046  1.793726182  2.091180241  1.757904594  2.349592856  1.882719113  1.103624418  0.99320049  1.894682147  2.570650235  1.535234438  2.260641617  1.334965174  2.530106675  2.093580134  2.517146936  2.078478573  2.360247069  2.45460046  3.177778083  1.823585316  1.985989242  0.787675502  2.721698412  1.371216695  3.399970862  2.184697775  2.496463075  1.912862876  0.447784502  1.410410992  1.400583521  0.963515852  2.48483257  0.952607959  1.415451982  1.717910896  1.982961932  1.646022857  2.481647595  2.068915841  1.805566027  2.058754498  2.286727737  2.152577943  1.031906709  2.156595861  2.787288469  1.804454104  2.538158493  2.041365608  1.866144881  1.783501034  2.458079445  0.185547521  1.122100523  2.71110631  1.715485601  2.347918285  1.650343983  1.628941334  2.470790492  2.977274531  3.031322329  1.699821714  0.801307974  1.392020532  1.611419091  0.834049454  2.288360703  1.514850777  2.003132196  2.186516689  1.849592257  2.664933322  1.407877357  1.451305589  1.783435224  1.834909246  3.059324064  1.70293411  2.38726752  2.605709013  2.271957367  1.111517702  2.639896955  2.480291657  1.812782403  3.058065677  3.375332701  0.748365714  2.680995286  2.295266715  1.982669783  1.375065707  2.372990459  2.217414395  1.610647456  2.568703653  2.070386483  1.121128589  1.903904124  1.323098462  1.811848198  2.509117542  2.406602428  1.424052701  1.168173378  1.443977512  2.449408346  0.559531063  2.322386546  2.394943058  0.764439429  2.111314717  1.251438027  2.683638324  2.431069498  3.346706067  2.001106704  1.431215002  1.619691457  3.405287001  1.9139626  1.741873621  2.094780756  2.090659049  3.029812758  2.0953666  2.64697087  2.332169013  1.641452976  1.825662791  1.502944891  0.899733136  0.566011321  1.96595216  2.650677689  3.078139384  3.200487755  1.236783463  2.133770145  1.81988244  1.376174645  1.372690059  2.342518284  1.338927333  2.326085501  2.470976765  1.55261182  1.306689762  2.6880796  2.437028885  1.492740178  2.838112442  2.79136286  1.886570999  1.266045153  2.55053455  1.960664733  0.852361969  2.628419795  1.267972963  1.745251697  2.846989397  1.229418193  2.485155248  1.272135612  1.627163263  1.059109078  0.524344688 | | low  low  low  high  low  low  low  high  low  low  high  low  low  low  low  high  low  low  high  low  low  low  high  low  high  low  low  low  high  high  high  high  high  low  low  high  high  low  low  low  high  high  low  low  high  high  high  high  high  high  low  low  high  low  high  low  low  low  low  high  low  high  low  high  high  high  high  high  high  high  low  high  low  high  low  high  high  high  high  low  low  low  low  high  low  low  low  high  low  high  high  low  high  high  high  low  high  high  low  high  high  low  low  high  low  low  high  low  high  low  low  high  high  high  low  low  low  low  low  high  low  high  high  low  high  low  low  low  low  high  low  high  high  high  low  high  high  low  high  high  low  high  high  high  low  high  high  low  high  high  low  low  low  low  high  high  low  low  low  high  low  high  high  low  high  low  high  high  high  high  low  low  high  high  low  high  high  high  high  high  high  low  low  low  low  low  high  high  high  high  low  high  low  low  low  high  low  high  high  low  low  high  high  low  high  high  low  low  high  high  low  high  low  low  high  low  high  low  low  low  low |
